# Causal link between metabolic related factors and osteoarthritis: a Mendelian randomization investigation

**DOI:** 10.3389/fnut.2024.1424286

**Published:** 2024-08-14

**Authors:** Kai Li, Yan Leng, Di Lei, Haojie Zhang, Minghui Ding, Wai Leung Ambrose Lo

**Affiliations:** ^1^Department of Rehabilitation Medicine, The First Affiliated Hospital, Sun Yat-sen University, Guangzhou, China; ^2^Guangdong Engineering and Technology Research Centre for Rehabilitation Medicine and Translation, The First Affiliated Hospital, Sun Yat-sen University, Guangzhou, China

**Keywords:** osteoarthritis, omega-3 fatty acids, omega-6 fatty acids, Mendelian randomization, genome-wide association study, fasting glucose

## Abstract

**Introduction:**

Metabolic syndrome (MetS) is significantly associated with osteoarthritis (OA), especially in MetS patients with blood glucose abnormalities, such as elevated fasting blood glucose (FG), which may increase OA risk. Dietary modifications, especially the intake of polyunsaturated fatty acids (PUFAs), are regarded as a potential means of preventing MetS and its complications. However, regarding the effects of FG, Omega-3s, and Omega-6s on OA, the research conclusions are conflicting, which is attributed to the complexity of the pathogenesis of OA. Therefore, it is imperative to thoroughly evaluate multiple factors to fully understand their role in OA, which needs further exploration and clarification.

**Methods:**

Two-sample univariable Mendelian randomization (UVMR) and multivariable Mendelian randomization (MVMR) were employed to examine the causal effect of metabolic related factors on hip OA (HOA) or knee OA (KOA). The exposure and outcome datasets were obtained from Open GWAS IEU. All cases were independent European ancestry data. Three MR methods were performed to estimate the causal effect: inverse-variance weighting (IVW), weighted median method (WMM), and MR-Egger regression. Additionally, the intercept analysis in MR-Egger regression is used to estimate pleiotropy, and the IVW method and MR-Egger regression are used to test the heterogeneity.

**Results:**

The UVMR analysis revealed a causal relationship between FG and HOA. By MVMR analysis, the study discovered a significant link between FG (OR = 0.79, 95%CI: 0.64∼0.99, p = 0.036) and KOA after accounting for body mass index (BMI), age, and sex hormone-binding globulin (SHBG). However, no causal effects of FG on HOA were seen. Omega-3s and Omega-6s did not have a causal influence on HOA or KOA. No significant evidence of pleiotropy was identified.

**Discussion:**

The MR investigation showed a protective effect of FG on KOA development but no causal relationship between FG and HOA. No causal effect of Omega-3s and Omega-6s on HOA and KOA was observed. Shared genetic overlaps might also exist between BMI and age, SHBG and PUFAs for OA development. This finding offers a novel insight into the treatment and prevention of KOA from glucose metabolism perspective. The FG cutoff value should be explored in the future, and consideration should be given to demonstrating the study in populations other than Europeans.

## Introduction

Osteoarthritis (OA) is a chronic joint disease with a high prevalence rate worldwide (1). It is among the leading musculoskeletal causes of impaired mobility in older adults (2). OA is characterized by the progressive degeneration of the joint cartilage and bone hyperplasia (3), which results in joint locking, swelling, instability, and pain. The most affected joints include the knees, hands, hips, and ankles. The quality of life of OA patients is often affected (4) due to difficulties in performing activities of daily living such as walking, standing, and going up and down stairs (5). Established risk factors for OA include age, body mass index (BMI), genetics, age, sex hormone, and diet (6, 7). Owing to the complex mechanism, effective treatment options to prevent the development of OA are limited despite extensive research efforts. Joint replacement surgery remains the only practical option for advanced OA cases. Consequently, there is an urgent need to identify the causal mechanism linking metabolic factors to OA and develop alternative management strategies. Recent studies have reported that patients with metabolic syndrome (MetS) are more susceptible to the occurrence of OA (8).

One indicator of MetS is abnormal fasting glucose (FG). Different views and research results exist on the relationship between FG and OA. One theory is that abnormal FG may increase the risk of osteoarthritis. The hyperglycemic state may lead to damage and inflammation of articular cartilage, thus promoting the development of OA. The Southeast European study found that high blood glucose was associated with knee lesions, and a 1.7% increase in blood glucose increased disease risk (9). A similar association was found in patients with hip osteoarthritis (HOA) (10). However, another view is that the relationship between abnormal FG and OA is unclear, or the relationship is affected by other factors, such as BMI. The Singapore study (11) suggests that diabetes or related medications may have a protective effect against knee osteoarthritis (KOA) (12). The researchers further found that hypoglycemic agents slowed the progression of KOA in patients only in obese diabetics (13). In addition, receiving insulin therapy can also delay the progression of knee arthritis (14). However, a study found that FG in men is only associated with OA in the hands, which are non-weight-bearing joints (15). To provide genetic information support and novel strategies for the early management and treatment of OA patients, the study selected healthy people who were relatively easy to control the confounders to investigate the causal relationship between FG and OA.

Nutritional supplements of polyunsaturated fatty acids (PUFAs), including omega-3 fatty acids (Omega-3s) and omega-6 fatty acids (Omega-6s), play a complex role in the occurrence and development of OA and MetS (16, 17). Fish oil, the main component of Omega-3s, which consists of eicosatetraenoic acid (EPA) and docosahexaenoic acid (DHA), has also been demonstrated to be beneficial in reducing inflammation (16). Besides, Omega-3s may reduce insulin resistance, regulate lipid metabolism, and reduce the risk of MetS (18). However, whether Omega-3s reduce OA development by regulating metabolism remains unclear. For another component, the role of Omega-6s in OA development needs to be more conclusive. Studies reported that arachidonic acid (AA), a component of Omega-6s, contributes to the production of prostaglandins and leukotrienes, which are associated with inflammation (19, 20). However, other studies demonstrated conflicting evidence that the increased intake of AA was not associated with inflammation (21, 22). Thus, considerable controversy still surrounds the overall effect of PUFAs on the immune system (23, 24). While increased PUFAs consumption has been correlated with a reduction of MetS, specific mechanisms remain unclear. Investigating the role of Omega-3s and Omega-6s in OA may lead to evidence-based dietary guidelines that aim to prevent the onset of OA in high-risk populations.

To date, observational clinical studies on OA often need more randomized design, which decreases the accuracy of the results due to the potential confounding factors. Available clinical trials tend to have the limitation of a small sample size, which leads to inconclusive findings (25). The present study employed Mendelian randomization (MR) analysis to address this issue and investigate the causal relationship between exposure and disease. MR analysis is a statistical method that relies on genetic variants as instrumental variables (IVs). It is a powerful tool that provides strong evidence for causality by exploiting the random allocation of genetic variants at conception, which is analogous to a natural experiment (26). It utilizes genome-wide association studies (GWAS) datasets to identify genetic variants strongly associated with the exposure of interest. These genetic variants are then applied as IVs to estimate the causal effect of metabolic related factors on OA. In the present study, FG, Omega-3s, and Omega-6s were the exposures of interest, and OA was the disease of interest. By using genetic variation as a research tool under large data volumes, MR minimizes the confounding variables that plague observational studies, providing a more robust and unbiased estimate of causality.

**FIGURE 1:**
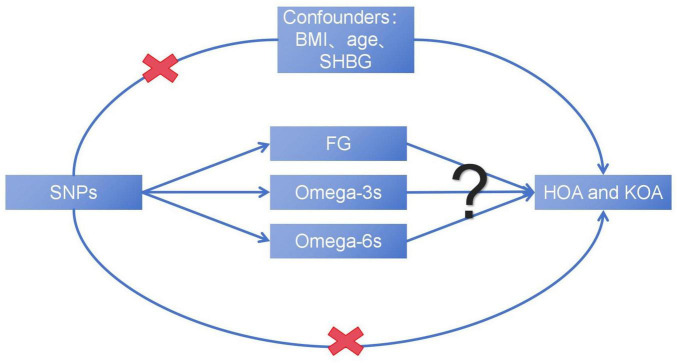
The basic assumption of a causal relationship between metabolic related factors and OA. UVMR, univariable Mendelian randomization; MVMR, multivariable Mendelian randomization.

In the study, we hypothesized that plasma levels of FG and PUFAs, including Omega-3s and Omega-6s, may affect the development of OA. This study aims to reveal the complex causal relationship between metabolic related factors and the advancing of OA (Figure 1), explore the potential impact of metabolic factors, and provide new insights into the treatment and prevention of OA.

## Materials and methods

### GWAS summary data source

**FIGURE 2:**
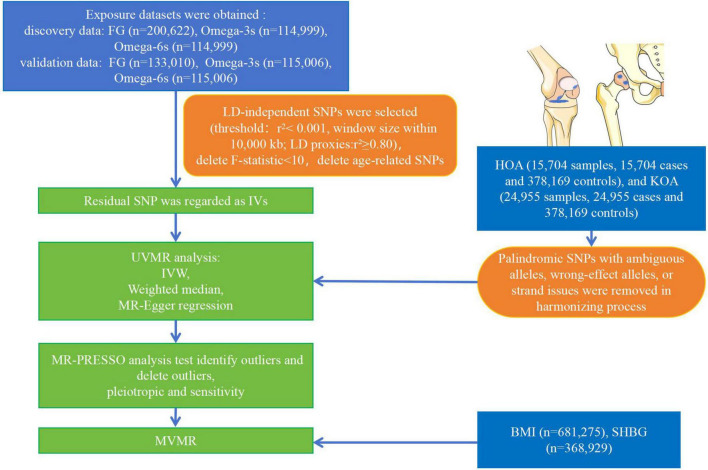
Flow chart of the analytical procedure. (LD, linkage disequilibrium; IVW, inverse variance weighting).

All cases were independent European ancestry derived from Open GWAS IEU^1^ (27). Relative information was searched on the GWAS website for “Fasting glucose,” “Omega-3 fatty acid,” and “Omega-6 fatty acid.” R 4.2.3 was used to obtain the raw data. The FG dataset (*n* = 200,622) (28–37) was collected from individuals without diabetes. The Omega-3s (*n* = 114,999) (38) and Omega-6s (*n* = 114,999) (38) were measured at baseline from individuals without stroke and coronary heart disease. Other datasets of FG (*n* = 133,010) (37), Omega-3s (*n* = 115,006) (39), and Omega-6s (*n* = 115,006) (39) were used to verify the above dataset. The FG validation dataset was collected from individuals without diabetes. The Omega-3s and Omega-6s validation datasets were measured baseline from individuals without coronary artery disease and type 2 diabetes. The study evaluated two phenotypes of OA. HOA (393,873 samples, including 15,704 cases and 378,169 controls) and KOA (403,124 samples, including 24,955 cases and 378,169 controls) were obtained from Open GWAS IEU (40). The data of HOA and KOA were obtained from individuals with primary HOA or KOA of radiographic Kellgren-Lawrence grade ≥ 2 or with clinical evidence of disease to a level requiring total joint replacement (41–43). Figure 2 illustrates the overall design of the study. The relevant review boards approved all studies, and the data were analyzed anonymously. Thus, no additional informed consent was required. Table 1 shows the demographic characteristics of all data.

**TABLE 1:** Demographic characteristics overview.

	Traits	Data sources	Sample size (case/control)	Ancestry
Exposure	FG Discovery	European Bioinformatics Institute	200,622	European
	FG Validation	Gibran et al.	133,010	European
	Omega-3s Discovery	Nightingale Health 2020	114,999	European
	Omega-3s Validation	European Bioinformatics Institute	115,006	European
	Omega-6s Discovery	Nightingale Health 2020	114,999	European
	Omega-6s Validation	European Bioinformatics Institute	115,006	European
	BMI	Gibran et al.	681,275	European
	SHBG	European Bioinformatics Institute	397,043	European
Outcome	HOA	European Bioinformatics Institute	393,873 (15,704/378,169)	European
	KOA	European Bioinformatics Institute	403,124 (24,955/378,169)	European

FG, fasting glucose; Omega-3s, omega-3 fatty acids; Omega-6s, omega-6 fatty acids; HOA, hip osteoarthritis; KOA, knee osteoarthritis; BMI, body mass index; SHBG, Sex hormone-binding globulin levels.

### Selection of genetic instrument variances

The single nucleotide polymorphisms (SNPs) were extracted from the GWAS dataset for FG, Omega-3s, and Omega-6s (*P* < 5 × 10^–8^). To ensure independence, the SNPs related to metabolic related factors and unrelated to linkage disequilibrium (LD) were clumped together (44) (*r*^2^ < 0.001, window size within 10,000 kb). LD proxies were estimated if SNPs were not identified in the outcome dataset (SNPs with high LD, *r*^2^ ≥ 0.80). Thresholds for minor allele frequency (MAF) > 0.3 were chosen to exclude palindromes SNPs. Only 1 SNP with LD was found in each analysis in this study. To identify weak IVs, the F-statistic for each SNP was calculated as follows: *F*-statistic = beta^2^/SE^2^ (beta is the effect estimate for the association of SNP with exposure, and SE is the standard error for the association) (45). F-statistic values less than 10 indicate weak IVs. Using LDtrait,^2^ the age-related SNPs were removed for Linkage Disequilibrium (46). To harmonize the effect between metabolic related factors and OA, ambiguous SNPs and missing data were removed, and palindromic SNPs with ambiguous alleles, wrong-effect alleles, or strand issues were harmonized.

### Statistical analysis

#### Mendelian randomization analyses

Univariable Mendelian randomization (UVMR) was used to evaluate the causal effect of OA-related metabolic factors. In this approach, genetic variants related to the exposure variables served as instrumental variables (IVs) to ascertain causal relationships. MR analysis is based on three core assumptions: (1) the IVs are strongly associated with the metabolic related factor; (2) the IVs are not related to other confounders; (3) the IVs influence the OA exclusively through the investigated factors. According to the first hypothesis, IVs are strongly associated with the exposure variable. The second and third assumptions are violated if IVs show horizontal pleiotropy, affecting outcomes through other causal pathways than exposure. Therefore, multiple sensitivity analyses were performed to detect and eliminate possible pleiotropic genetic variants, as detailed in the methods. The MR method can mitigate the influence of unmeasured confounding factors, thereby enabling more robust causal inference. Three MR methods were performed to estimate the causal effect of metabolic related factors for OA, mainly including inverse-variance weighting (IVW), weighted median method (WMM), and MR-Egger regression (47, 48). IVW estimated the causal effect based on the assumption that all the IVs listed are valid, which was regarded as the primary outcome. The WMM and MR-Egger regression were applied to complement IVW estimates. If the forecast of these methods is inconsistent, the threshold for *p*-value < 0.05 was set.

The effect of IVs was estimated by a 2-stage least-squares regression (2SLS). The UVMR results were presented as odds ratio (OR) and 95% confidence intervals (CIs), which are regarded as the change of OA for each additional standard deviation (SD) in metabolic related factors. The “TwoSampleMR” packages performed the MR method in R 4.2.3 (49).

In addition to UVMR, MVMR (50) was applied to investigate the causal effect of metabolic related factors on OA. Genetic associations between SNPs and BMI were derived from 681,275 European ancestry (51). Genetic associations between SNPs and SHBG (52) were obtained from 368,929 European ancestry. Both data, derived from GWAS, are typically obtained from multiple independent but rigorously screened research for health-related projects. Multivariable Mendelian randomization (MVMR) was applied to examine the effect of metabolic related factors independent of the confounding effect of BMI, age, and sex hormone-related factors like SHBG. The screening condition of SNPs is the same as UVMR.

#### Pleiotropic and sensitivity analysis

The intercept analysis in MR-Egger regression was used to estimate the horizontal and directional pleiotropy that contributed to the incorrect effect of MR analysis. In MR-PRESSO analysis, unexpected SNPs contributing to the increase of heterogeneity were removed. The IVW method and MR-Egger regression were used to test the heterogeneity, and Cochran’s Q statistic was used to evaluate the heterogeneity. P-value < 0.05 was set as significant heterogeneity. In addition, leave-one-out sensitivity analysis was adopted to recognize the potential influential SNPs. Funnel plots were also performed to evaluate the heterogeneity among SNPs.

## Results

### The causal effect of metabolic related factors on hip osteoarthritis

**FIGURE 3:**
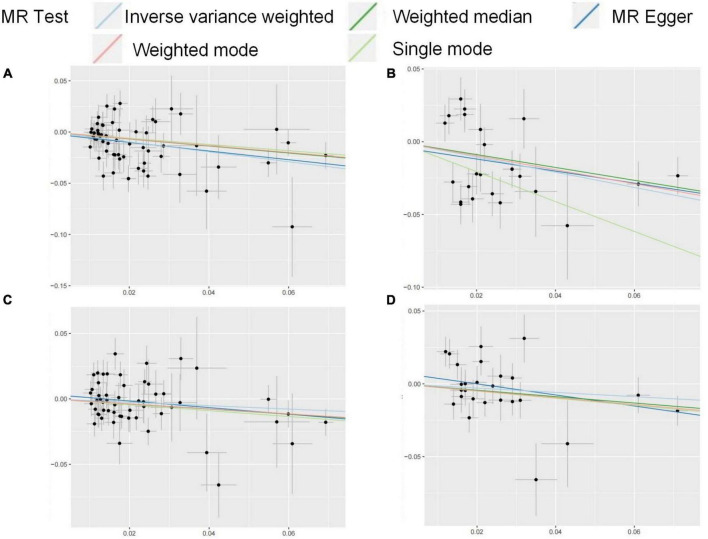
**(A–D)** Funnel plots for MR analysis between FG on OA. The x-coordinate is the effect size, and the y-coordinate is the standard error. **(A)** Funnel plot for FG discovery and HOA; **(B)** funnel plot for FG validation and HOA; **(C)** funnel plot for FG discovery and KOA; **(D)** funnel plot for FG validation and KOA.

**FIGURE 4:**
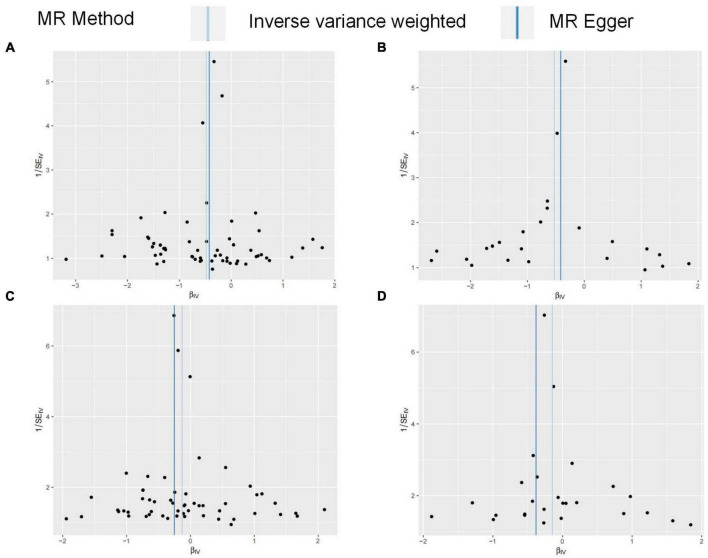
The graph of MR results in this study. The broken line represents the relationship between exposure and outcome in UVMR. The solid line represents the relationship between exposure and outcome in MVMR.

FG was found to be negatively correlated with HOA in UVMR. The IVW method indicated that every 1SD increase in the FG reduced the risk of HOA by 38% (OR: 0.62, 95%CI: 0.50∼0.76) (Table 2). WMM (OR: 0.71, 95%CI: 0.55∼0.93) and MR-Egger regression (OR: 0.66, 95%CI: 0.45∼0.96) (Table 2) showed similar results. The calculated causal effect of FG on HOA was consistent across the five methods (Figure 3A). Six week-IVs (rs896854, rs2657879, rs4760278, rs17270243, rs12898997, rs39713) were identified by F-statistic and removed. One age-related IV (rs11708067) was removed. Three SNP outliers (rs1260326, rs12898997, rs7012637) were observed and removed based on the MR-PRESSO result. Though Cochran’s Q test for IVW (*P* < 0.001) and MR-Egger (*P* < 0.001) showed heterogeneity among SNPs, sensitivity analysis did not review any pleiotropy (*P* = 0.717). The funnel plot was presented in Figure 4A, and its symmetry indicated no violation of the IV assumption. No single SNP strongly influences the overall effect of FG on HOA in the leave-one-out analysis (Supplementary Figure 3A). The forest plot is presented in Supplementary Figure 6A. When the unified method of analysis was performed using another FG data, a consistent result of the causal relationship between FG and HOA by IVW (OR: 0.59, 95%CI: 0.43∼0.82), by WMM (OR: 0.64, 95%CI: 0.48∼0.86), by MR-Egger (OR: 0.66, 95%CI: 0.35∼1.26). The calculated causal effect of FG on HOA was consistent across the five methods (Figure 3B). F-statistic identified no week-IVs. Five age-related IVs (rs6072275, rs7903146, rs174576, rs11708067, rs983309) was removed. Two SNP outliers (rs1260326, rs6943153) were observed and removed based on the MR-PRESSO result. Cochran’s *Q* test for IVW (*P* < 0.001) and MR-Egger (*P* < 0.001) showed heterogeneity among SNPs. Sensitivity analysis did not review any pleiotropy (*P* = 0.704). The funnel plot was presented in Figure 4B, and its symmetry indicated no violation of the IV assumption. No single SNP strongly influences the overall effect of FG on HOA in the leave-one-out analysis (Supplementary Figure 3B). The forest plot is presented in Supplementary Figure 6B.

**TABLE 2:** Causal effect estimates of metabolic related factors on OA after univariable MR result.

Exposure	Outcome	SNPs number	IVW method		Weighted median		MR-Egger regression	
			OR (95%CI)	*P*-value	OR (95%CI)	*P*-value	OR (95%CI)	*P*-value
FG Discovery	HOA	60	0.62 (0.50∼0.76)	<0.001	0.71 (0.55∼0.93)	0.012	0.66 (0.45∼0.96)	0.034
FG Validation	HOA	24	0.59 (0.43∼0.82)	0.001	0.64 (0.48∼0.86)	0.003	0.66 (0.35∼1.26)	0.222
Omega-3s Discovery	HOA	37	0.98 (0.88∼1.10)	0.770	1.05 (0.91∼1.22)	0.522	0.90 (0.73∼1.11)	0.350
Omega-3s Validation	HOA	37	0.99 (0.89∼1.10)	0.864	1.05 (0.89∼1.23)	0.592	0.97 (0.80∼1.18)	0.752
Omega-6s Discovery	HOA	39	0.90 (0.80∼1.00)	0.057	0.97 (0.83∼1.14)	0.709	0.90 (0.71∼1.14)	0.403
Omega-6s Validation	HOA	46	0.96 (0.88∼1.06)	0.432	1.00 (0.87∼1.15)	0.990	0.95 (0.79∼1.14)	0.584
FG Discovery	KOA	55	0.88 (0.75∼1.03)	0.113	0.82 (0.68∼1.00)	0.049	0.78 (0.58∼1.04)	0.100
FG Validation	KOA	26	0.86 (0.70∼1.06)	0.166	0.80 (0.65∼1.00)	0.049	0.68 (0.46∼1.03)	0.078
Omega-3s Discovery	KOA	37	0.90 (0.83∼0.98)	0.018	0.89 (0.79∼1.01)	0.062	0.93 (0.79∼1.10)	0.391
Omega-3s Validation	KOA	39	0.92 (0.84∼1.00)	0.048	0.92 (0.82∼1.03)	0.161	0.99 (0.84∼1.16)	0.881
Omega-6s Discovery	KOA	46	0.90 (0.82∼0.98)	0.015	0.89 (0.80∼0.99)	0.030	0.84 (0.71∼1.00)	0.051
Omega-6s Validation	KOA	46	0.92 (0.84∼1.00)	0.044	0.89 (0.80∼0.99)	0.033	0.83 (0.71∼0.98)	0.033

FG, fasting glucose; Omega-3s, omega-3 fatty acids; Omega-6s, omega-6 fatty acids; HOA, hip osteoarthritis; KOA, knee osteoarthritis; BMI, body mass index; SHBG, Sex hormone-binding globulin levels; OR, odds ratio.

There was no relationship between Omega-3s and HOA by IVW method (OR: 0.98, 95%CI: 0.88∼1.10), WMM (OR: 1.05, 95%CI: 0.91∼1.22) and the MR-Egger regression (OR: 0.90, 95%CI: 0.73∼1.11) (Table 2). All conditional F statistics of exposure were large, indicating no weak IVs exist. After screening by MR-PRESSO, one SNP (rs16940904) with significant deviations was removed, which did not change the causal estimates. Eleven age-related IVs (rs1260326, rs62466318, rs964184, rs10096633, rs112875651, rs12914626, rs157592, rs2072114, rs2269928, rs58542926, rs629301) was removed. Cochran’s Q test for IVW (*P* = 0.134) and MR-Egger (*P* = 0.133) showed no heterogeneity among SNPs. No pleiotropy was found in sensitivity analysis (*P* = 0.357). Besides, the symmetry of the funnel plot indicated no violation of the IV assumption (Supplementary Figure 1A). In the leave-one-out-analysis, no single SNP strongly influenced the overall effect of Omega-3s on HOA (Supplementary Figure 4A). The forest plot is presented in Supplementary Figure 7A. When analyzing other Omega-3s data, no causal relationship between Omega-3s and HOA was identified by IVW (OR: 0.99, 95%CI: 0.89∼1.10), by WMM (OR: 1.05, 95%CI: 0.89∼1.23), by MR-Egger (OR: 0.97, 95%CI: 0.80∼1.18). F-statistic identified no week-IVs. Thirteen age-related IVs (rs1260326, rs312939, rs62466318, rs964184, rs58542926, rs10096633, rs112875651, rs157592, rs2072114, rs2269928, rs633695, rs660240, rs9987289) was removed. One SNP outlier (rs16940904) was observed and removed based on the MR-PRESSO result. Cochran’s Q test for IVW (P = 0.307) and MR-Egger (P = 0.270) showed no heterogeneity among SNPs. Sensitivity analysis did not review any pleiotropy (P = 0.788). The funnel plot was presented in Supplementary Figure 1B, and its symmetry indicated no violation of the IV assumption. No single SNP strongly influences the overall effect of Omega-3s on HOA in the leave-one-out analysis (Supplementary Figure 4B). The forest plot is presented in Supplementary Figure 7B.

There was no causal relationship between Omega-6s and HOA in UVMR. From MR analysis, the IVW result suggested no relationship between Omega-6s and HOA (OR: 0.90, 95%CI: 0.80∼1.00) (Table 2). Similar results were also obtained by WMM (OR: 0.97, 95%CI: 0.83∼1.14) (Table 2) and MR-Egger regression (OR: 0.90, 95%CI: 0.71∼1.14) (Table 2). The result of Omega-6s on HOA calculated by the five methods was consistent (Supplementary Figure 2A). All conditional F statistics were larger than ten, which indicated no weak IVs. Nineteen age-related SNPs (rs1002687, rs1065853, rs1081105, rs112875651, rs11789603, rs142158911, rs1461729, rs1800961, rs183130, rs2378390, rs2740488, rs34121855, rs4439799, rs4704210, rs633695, rs6547409, rs77960347, rs9304381, rs964184) were removed. After removing 2 SNPs (rs3817335 and rs6934962) selected by the MR-PRESSO method, Cochran’s Q test for IVW (P = 0.159) and MR-Egger (P = 0.133) indicated no heterogeneous SNPs. No pleiotropy was observed in sensitivity analysis (P = 0.955). The symmetry of the funnel plot also indicated no violation of the IV assumption (Supplementary Figure 2A). The leave-one-out analysis did not reveal any single SNP strongly driving the overall effect of Omega-6s on HOA (Supplementary Figure 5A). The forest plot is presented in Supplementary Figure 8A. When using the same method to analyze other Omega-6s data, no causal relationship between Omega-6s and HOA was identified by IVW (OR: 0.96, 95%CI: 0.88∼1.06), by WMM (OR: 1.00, 95%CI: 0.87∼1.15), by MR-Egger (OR: 0.95, 95%CI: 0.79∼1.14). F-statistic identified no week-IVs. Thirteen age-related IVs (rs4704210, rs1260326, rs34121855, rs672889, rs964184, rs58542926, rs1065853, rs1081105, rs112875651, rs3764261, rs602633, rs633695, rs2126259) were observed and removed. Three SNP outliers (rs3817335, rs6934962, rs74747585) were observed and removed based on the MR-PRESSO result. Cochran’s Q test for IVW (P = 0.363) and MR-Egger (P = 0.326) showed no heterogeneity among SNPs. Sensitivity analysis did not review any pleiotropy (P = 0.856). The funnel plot was presented in Supplementary Figure 2B, and its symmetry indicated no violation of the IV assumption. No single SNP strongly influences the overall effect of Omega-6s on HOA in the leave-one-out analysis (Supplementary Figure 5B). The forest plot is presented in Supplementary Figure 8B.

### The causal effect of metabolic related factors on knee osteoarthritis

There was no relation between FG and KOA by IVW method (OR: 0.88, 95%CI: 0.75∼1.03), the MR-Egger regression (OR: 0.82, 95%CI: 0.68∼1.00), though WMM indicated significant (OR: 0.78, 95%CI: 0.58∼1.04) (Table 2). The result of FG on KOA was consistent across these methods (Figure 3C). After F-statistics evaluation, six weak IVs (rs896854, rs2657879, rs4760278, rs17270243, rs12898997, rs39713) were identified and removed. After screening by the MR-PRESSO method, five SNPs with bias (rs1057394, rs10974438, rs1260326, rs12898997, rs3842753) were removed. Five age-related SNPs (rs17265513, rs7903146, rs174583, rs11708067) were removed. Cochran’s Q test revealed heterogeneity in SNPs for IVW (P = 0.004) and MR-Egger (P = 0.004) methods, suggesting that there may be confounding factors. The symmetry of the funnel plot suggested no violation of the IV assumption (Figure 4C). No pleiotropy was identified in sensitivity analysis (P = 0.336). Leave-one-out analysis showed that no single SNP strongly influenced the overall effect of FG on KOA (Supplementary Figure 3C). The forest plot is presented in Supplementary Figure 6C. When using the same method to analyze other FG data, no causal relationship between FG and KOA was identified by IVW (OR: 0.86, 95%CI: 0.70∼1.06), by WMM (OR: 0.80, 95%CI: 0.65∼1.00), by MR-Egger (OR: 0.68, 95%CI: 0.46∼1.03). The result of FG on KOA was consistent across these methods (Figure 3D). F-statistic identified no week-IVs. Two age-related IVs (rs174576, rs11708067) were observed and removed. Three SNP outliers (rs10814916, rs1260326, rs7903146) were observed and removed based on the MR-PRESSO result. Cochran’s Q test for IVW (P = 0.010) and MR-Egger (P = 0.016) showed heterogeneity among SNPs. Sensitivity analysis did not review any pleiotropy (P = 0.203). The funnel plot was presented in Figure 4D, and its symmetry indicated no violation of the IV assumption. No single SNP strongly influences the overall effect of FG on KOA in the leave-one-out analysis (Supplementary Figure 3D). The forest plot is presented in Supplementary Figure 6D.

The result showed that Omega-3s were negatively correlated with KOA in UVMR. The result of the IVW method suggested that a 1SD increase in Omega-3s resulted in a 10% reduction in KOA risk (OR: 0.90, 95%CI: 0.83∼0.98) (Table 2). There was no causal relationship between Omega-3s and KOA in WMM (OR: 0.89, 95%CI: 0.79∼1.01) and MR-Egger regression (OR: 0.93, 95%CI: 0.79∼1.10) (Table 2). All conditional F statistics were greater than 10, indicating the absence of weak IVs of exposure. Evelen age-related SNPs (rs1260326, rs62466318, rs964184, rs10096633, rs112875651, rs12914626, rs157592, rs2072114, rs2269928, rs58542926, rs629301) were removed. After excluding one outlier SNPs (rs6601924) identified by MR-PRESSO, Cochran’s Q test indicated no heterogeneity between SNPs for both IVW (P = 0.155) and MR-Egger (P = 0.134) methods. Besides, the screening did not change the causal estimates. The symmetry of the funnel plot suggested no violation of the IV assumption (Supplementary Figure 1C). No pleiotropy was found in sensitivity analysis (P = 0.669). No single SNP strongly influenced the overall effect in the leave-one-out analysis (Supplementary Figure 4C). The forest plot is presented in Supplementary Figure 7C. When using the same method to analyze other Omega-3s data, negatively causal relationship between Omega-6s and KOA was identified by IVW (OR: 0.92, 95%CI: 0.84∼1.00), while no causal relationship by WMM (OR: 0.92, 95%CI: 0.82∼1.03), by MR-Egger (OR: 0.99, 95%CI: 0.84∼1.16). F-statistic identified no week-IVs. Ten age-related IVs (rs157592, rs1260326, rs62466318, rs964184, rs58542926, rs10096633, rs112875651, rs2072114, rs2269928, rs9987289) were observed and removed. Two SNP outliers (rs6601924, rs6129624) were observed and removed based on the MR-PRESSO result. Cochran’s Q test for IVW (P = 0.052) and MR-Egger (P = 0.056) showed no heterogeneity among SNPs. Sensitivity analysis did not review any pleiotropy (P = 0.295). The funnel plot was presented in Supplementary Figure 1D, and its symmetry indicated no violation of the IV assumption. No single SNP strongly influences the overall effect of Omega-3s on KOA in the leave-one-out analysis (Supplementary Figure 4D). The forest plot is presented in Supplementary Figure 7D.

The results of the genetic analysis suggested that Omega-6s have a negative association with KOA. The IVW result suggested that a 1SD increase of Omega-6s was associated with a 10% reduction in the risk of KOA (OR: 0.90, 95%CI: 0.82∼0.98) (Table 2). Similar results were obtained in the WMM (OR: 0.89, 95%CI: 0.80∼0.99) (Table 2). MR-Egger regression had no causal relationship (OR: 0.84, 95%CI: 0.71∼1.00) (Table 2). All conditional F statistics were greater than 10, indicating the absence of weak IVs. Thirteen age-related SNPs (rs4704210, rs1260326, rs34121855, rs672889, rs964184, rs1065853, rs1081105, rs112875651, rs12740374, rs183130, rs58542926, rs633695, rs1461729) were removed. After removing one SNPs (rs2378390) that showed significant bias according to MR-PRESSO results, Cochran’s Q test IVW (P = 0.031) and MR-Egger (P = 0.031) indicated heterogeneity among SNPs. The symmetry of the funnel plot suggested no violation of the IV assumption (Supplementary Figure 2C). No pleiotropy was found in the sensitivity analysis (P = 0.385). The leave-one-out analysis showed that no single SNP was driving the overall effect of Omega-6s on KOA (Supplementary Figure 5C). The forest plot is presented in Supplementary Figure 8C. When using the same method to analyze other Omega-6s data, negatively causal relationship between Omega-6s and KOA was identified by IVW (OR: 0.86, 95%CI: 0.70∼1.06), by WMM (OR: 0.80, 95%CI: 0.65∼1.00), by MR-Egger (OR: 0.68, 95%CI: 0.46∼1.03). F-statistic identified no week-IVs. Thirteen age-related IVs (rs4704210, rs1260326, rs34121855, rs672889, rs964184, rs58542926, rs1065853, rs1081105, rs112875651, rs3764261, rs602633, rs633695, rs2126259) were observed and removed. Two SNP outliers (rs2378390, rs117488242) were observed and removed based on the MR-PRESSO result. Cochran’s Q test for IVW (*P* = 0.013) and MR-Egger (*P* = 0.021) showed heterogeneity among SNPs. Sensitivity analysis did not review any pleiotropy (*P* = 0.144). The funnel plot was presented in Supplementary Figure 2D, and its symmetry indicated no violation of the IV assumption. No single SNP strongly influences the overall effect of Omega-6s on KOA in the leave-one-out analysis (Supplementary Figure 5D). The forest plot is presented in Supplementary Figure 8D.

### Multivariable MR analyses

The results of the MVMR revealed that FG had a protective effect on KOA (IVW OR: 0.79, 95%CI: 0.64∼0.99, *P* = 0.036), while without effect on HOA (IVW OR: 0.79, 95%CI: 0.61∼1.02, *P* = 0.068) was observed. After deleting the age-related SNPs and adjusting for BMI and SHBG, circulating Omega-3s were not associated with the occurrence of HOA (*P* = 0.774) and KOA (*P* = 0.473). After adjustment, circulating Omega-6s were unrelated to HOA (*P* = 0.494) and KOA (*P* = 0.143). Additionally, the risk of BMI was found to be associated with a higher risk of OA in both hip joint (IVW OR: 1.56, 95%CI: 1.33∼1.82, *P* < 0.001) and knee joint (IVW OR: 1.86, 95%CI: 1.63∼2.11, *P* < 0.001). SHBG was unrelated to HOA (*P* = 0.675) and KOA (*P* = 0.235). MVMR results are shown in Supplementary Table 1.

## Discussion

**FIGURE 5:**
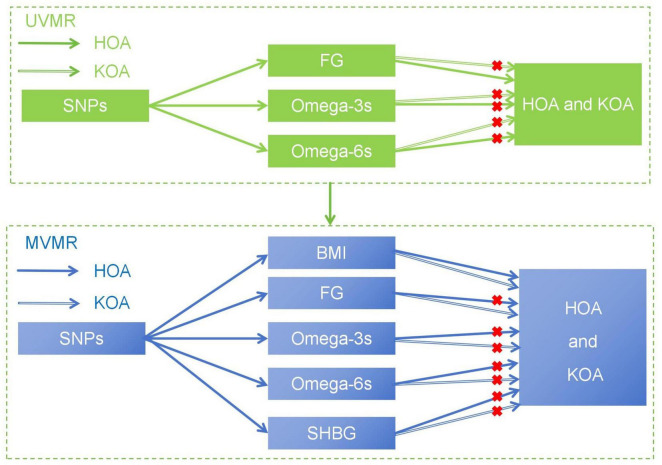
**(A–D)** Scatter plots for the MR result of the causal effect of FG on OA. MR methods included IVW, Weighted median, MR-Egger, Weighted mode, and Simple mode methods. The x-coordinate is the effect of SNPs on the exposure, and the y-coordinate is the effect of SNPs on the outcome. **(A)** effect of FG discovery on HOA; **(B)** effect of FG validation on HOA; **(C)** effect of FG discovery on KOA; **(D)** effect of FG validation on KOA.

This study used the metabolic factors datasets from GWAS to investigate the causal impact of metabolic related factors on OA in weight-bearing joints (hip and knee). Our findings indicate that after adjusting for BMI, age, and SHBG, FG is negatively associated with the risk of KOA while irrelated with HOA. Omega-3s and Omega-6s are irrelated with the occurrence of OA. The MR results in this study were shown in Figure 5.

The study discovered that FG is correlated with the development of HOA in UVMR while uncorrelated with the occurrence of KOA. After adjustment for BMI and SHBG in MVMR, FG is negatively related to the risk of KOA while irrelated to the risk of HOA. The above results indicate that FG plays a protective role in KOA development, while there is no effect of FG on HOA. The clinical subtype of metabolic syndrome-associated osteoarthritis (MetS-OA) underscores the importance of MetS as a risk factor for OA (53, 54). MetS is characterized by insulin resistance, obesity, atherogenic dyslipidemia, and hypertension, among other characteristics (55). This study observed a causal link between FG levels and a reduced risk of KOA, while there was no relationship between FG and HOA. This study result is inconsistent with previous studies, which proposed that FG could be a risk factor for MetS-related OA due to its pro-inflammatory properties. Preclinical trials have shown a macrophage shift toward the M1 phenotype within the synovial and adipose tissues in diet-induced OA (56). A significant contributor to this inflammatory response is the presence of advanced glycation end-products (AGEs) that originate from chronic hyperglycemia. Receptors for AGEs (RAGEs) expressed on macrophages recognize these molecules, resulting in M1 polarization and upregulation of pro-inflammatory cytokines such as TNF and IL-1β via NF-kB activation (57). Higher AGE levels in the subchondral bone were observed in OA patients with diabetes than in those without diabetes (58, 59), which appears to support the role of FG in OA development. Hyperglycemia also elevates oxidative stress by overproducing nitric oxide and reactive oxygen species (60), which further promote articular chondrocyte apoptosis (61) and articular cartilage destruction (62). However, the conflicting findings are consistent with a long-term follow-up study. Clinical data from a 32-year follow-up study reported that MetS did not increase KOA risk, and elevated levels of FG were associated with a lower OA risk (12). High glucose levels can induce endothelial cells to transdifferentiate into chondrocyte-like cells through a process known as endothelial-to-mesenchymal transition, which facilitates chondrocyte proliferation (63). Given that chondrocytes are a significant component that forms extracellular matrix within the cartilage (64), the glucose metabolism pathway may have a beneficial influence on the pathogenesis of OA. This notion is further supported by an animal study that reported that glucose supplementation in mice models could reverse fasting-induced cellular immunosuppression (65). Therefore, the study on glucose metabolism of OA should be based on the analysis of the level of FG in healthy people and diabetic groups, respectively. For healthy people, more attention should be paid to blood glucose stability. Blood sugar stability also reflects physical activity to some extent (66). The findings of this study advise adding to the phenotype of fasting glucose studies in OA and the potential beneficial effects of glucose stability in non-diabetic individuals. Previous studies (67, 68) suggested epigenetic, pathophysiological, and biomechanical differences between HOA and KOA. A recent study used MR analysis to discover ten circulating proteins, including SHBG, which is causally associated with traits associated with osteoarthritis (OA) (69). However, our study found no link between SHBG and OA in MVMR. The causal effect of FG on HOA was correlated with BMI and SHBG, but the impact on KOA was independent of BMI and SHBG. This finding is consistent with a previous study that found no correlation between SHBG and the risk of hip replacement, while an association between SHBG and the risk of hip replacement. Future studies should consider the relationship between the circulating levels of all endogenous hormones and OA.

The study reaffirmed that BMI is a risk factor for HOA and KOA, and this result is similar to the results of previous studies (70, 71). A dose-response meta-analysis has shown that the risk of KOA increases almost exponentially with increasing BMI (41). One crucial factor is that being overweight places a significant mechanical load on the knee (42), which can lead to alterations in the composition and structure of articular cartilage (43). However, abnormal weight bearing could not solely explain the occurrence of OA since OA of the hands, a non-load-bearing joint, is also associated with obesity (44). Given that metabolic syndrome is often associated with obesity, it is plausible that both mechanical overloading and systemic factors contribute to the initiation and exacerbation of OA in weight-bearing joints. In this study, BMI was selected for multivariate analysis to determine whether other components combined with BMI impacted OA development.

The study’s findings indicated that once the metabolic factor was adjusted for BMI, age, and SHBG, the causal link between OA, Omega-3s, and Omega-6s disappeared. Omega-3s and omega-6s are the two main types of PUFAs that play potential roles in osteoarthritis (72). Existing research has shown that the cancellous bone matrix from the subchondral bone of OA patients contains higher levels of PUFAs than healthy controls (73). Omega-3s is recognized for its anti-inflammatory properties, whereas Omega-6s have pro-inflammatory effects. The mechanism for the anti-inflammatory effect of Omega-3s is related to its ability to reduce the production of pro-inflammatory prostaglandins and leukotrienes derived from arachidonic acid (AA) (74). However, the extent of their efficacy in modulating inflammatory processes remains uncertain. Omega-3s engage in enzymatic competition with Omega-6s in eicosanoid synthesis, decreasing pro-inflammatory eicosanoid production (75). It can also modulate the activation of macrophages and T cells to reduce inflammatory response (72). Preclinical findings support Omega-3s as a prospective treatment for OA, and human research also supports that Omega-3s may be effective. Still, additional investigation is needed to establish the most effective treatment protocols (16). Research to date suggests that Omega-3s improves functional and pain levels in patients with OA (76), but further investigation is required to understand their physiological benefits. This study proves that Omega-3s are not associated with OA development but may be effective in symptom management, such as pain relief and improved function.

Omega-6s play a role in the production of AA (76), and a positive correlation between Omega-6s and OA was previously observed (77). Elevated lipid levels and Omega-6s have also been found in OA bone (78). However, controversial findings indicated that AA intake at doses up to 1000-1500mg per day may not adversely affect immune response or inflammation. A daily AA intake of 1000mg might even reduce inflammatory markers such as IL-6 and increase prostaglandin E2 (PGE2), particularly in older individuals (55). Based on the extensive genetic data from our large sample study, our findings suggest no causal link between Omega-6s and OA after adjusting for BMI, age, and SHBG. Some studies have speculated that the ratio between Omega-6s and Omega-3s, rather than the quantity of either fatty acids, minimizes oxidative stress, subsequently influencing OA occurrence (79, 80).

## Strengths and limitations

The study’s strength is the use of MR Analysis, which reduces the possibility of reverse causality bias and effectively controls for confounding factors, thus improving the accuracy of the causality assessment between exposure and outcome. The reliability of the findings was enhanced by multivariate analysis of BMI, age, and SHBG. In addition, sensitivity analysis demonstrated the robustness of the MR Results, further ensuring the reliability of the study conclusions.

The study has several limitations that need to be acknowledged. Firstly, the samples were limited to the European population, and more research is required to assess their applicability to other ethnic groups. In the future, data from different ethnic groups can be collected to explore the specific mechanism of the association between MetS and osteoarthritis among various ethnic groups. Secondly, while studies have highlighted the role of metabolic factors in knee and hip osteoarthritis, the relevance of hand osteoarthritis needs to be clarified. Future research should extend the investigation to include hand OA and assess whether similar relationships are observed. Thirdly, an in-depth subgroup analysis was impossible due to the lack of detailed demographic information and study patient records. Due to the lack of detailed original data, this study could not identify the FG truncation value most negatively correlated with KOA. Large-scale clinical trials can be conducted to collect more detailed data on KOA patients to verify and correct the FG truncation value. Finally, in this study, UVMR and MVMR were used for the study by adding factors related to BMI, age, and SHBG could not completely exclude other factors affecting OA. In the future, mediated MR could be used to investigate further whether mediating factors influence the development of OA.

## Conclusion

The current study’s findings enhance our comprehension of the potential impact of metabolic factors on the onset of osteoarthritis (OA). While no causal link was established between polyunsaturated fatty acids (PUFAs) and OA, their effects may be modulated by the ratio of Omega-3 to Omega-6s, elevated body mass index (BMI), or sex hormone-binding globulin (SHBG) levels. Furthermore, there may be shared genetic predispositions between BMI and aging, SHBG, and PUFAs that contribute to OA development. The above factors can be further analyzed and discussed in the future. Interestingly, fasting glucose (FG) appears to have a protective role against knee OA (KOA) but does not significantly influence hip OA (HOA), providing a fresh perspective on the therapeutic and preventive strategies for KOA from a glucose metabolism angle. Future research should delve into determining an optimal FG cutoff value and replicate these findings across diverse populations beyond Europeans to ensure global applicability.

## Data availability statement

The datasets presented in this study can be found in online repositories. The names of the repository/repositories and accession number(s) can be found below: https://gwas.mrcieu.ac.uk/, ebi-a-GCST90002232; met-d-Omega_3; met-d-Omega_6; ebi-a-GCST007091; ebi-a-GCST007090; ieu-b-114; ebi-a-GCST90092931; ebi-a-GCST90092933.

## Ethics statement

Written informed consent was obtained from the individual(s) for the publication of any potentially identifiable images or data included in this article.

## Author contributions

KL: Conceptualization, Data curation, Investigation, Methodology, Writing−original draft. YL: Writing−original draft, Conceptualization, Data curation, Investigation, Methodology. DL: Writing−original draft, Conceptualization, Data curation, Formal analysis. HZ: Conceptualization, Data curation, Formal analysis, Writing−original draft. MD: Funding acquisition, Project administration, Resources, Supervision, Validation, Visualization, Writing−review and editing. WL: Funding acquisition, Project administration, Resources, Supervision, Validation, Visualization, Writing−review and editing.

## Footnotes

1
https://gwas.mrcieu.ac.uk/

2
https://ldlink.nih.gov/?tab=ldtrait
