# Supplementary material for: Causal link between metabolic related factors and osteoarthritis: a Mendelian randomization investigation
Source: Front Nutr. 2024 Aug 14;11:1424286. doi: 10.3389/fnut.2024.1424286 (PMC11349640; doi:10.3389/fnut.2024.1424286)
Supplement: Supplementary file 1 [file Data_Sheet_1.doc]

Supplementary Material

# 1.1 Supplementary Figures


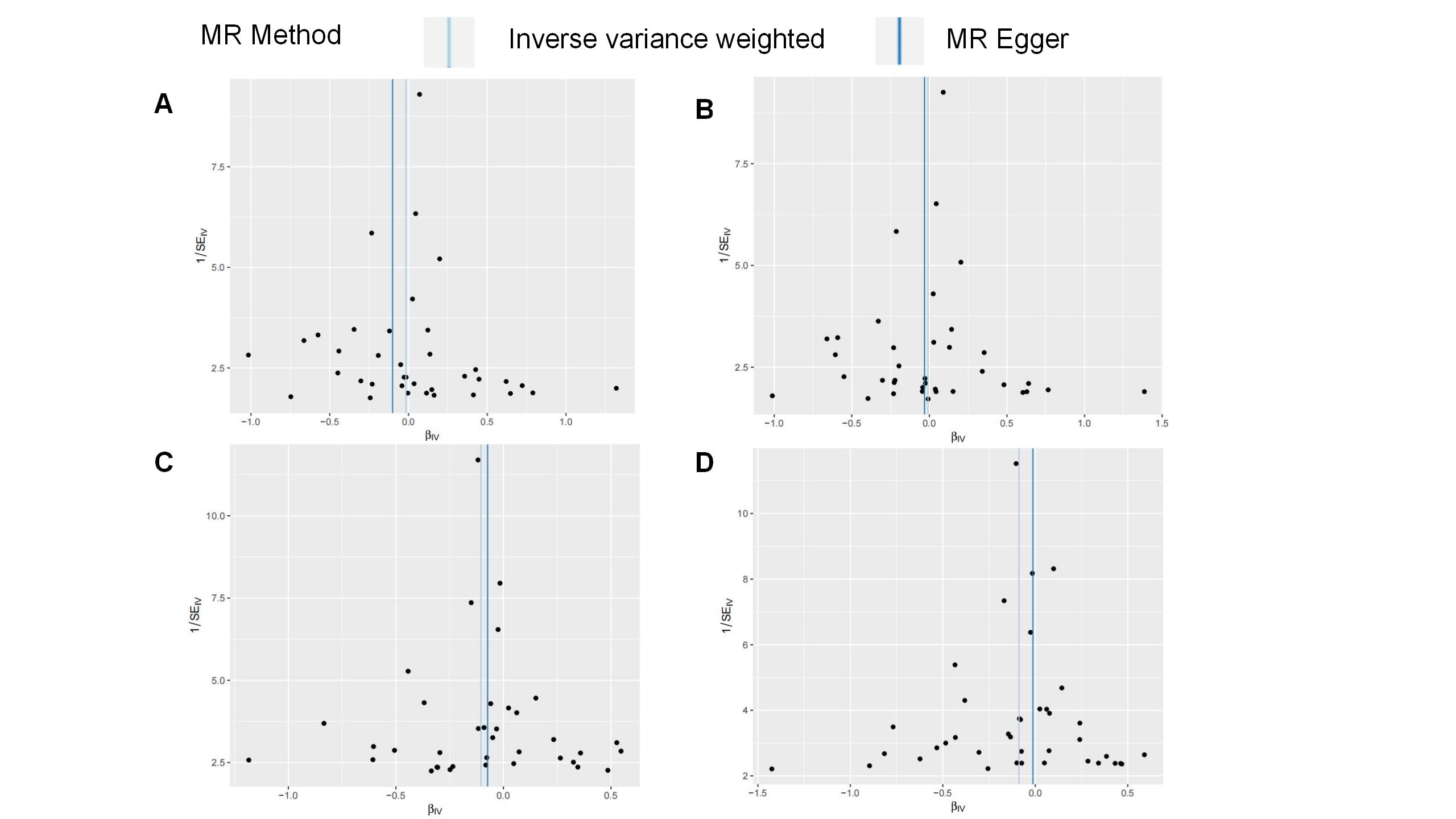


**Supplementary** **FIGURE 1.** (A-D) Funnel plots for MR analysis between Omega-3s on OA. The x-coordinate is the effect size, and the y-coordinate is the standard error. (A) funnel plot for Omega-3s discovery and HOA; (B) funnel plot for Omega-3s validation and HOA; (C) funnel plot for Omega-3s discovery and KOA; (D) funnel plot for Omega-3s validation and KOA.


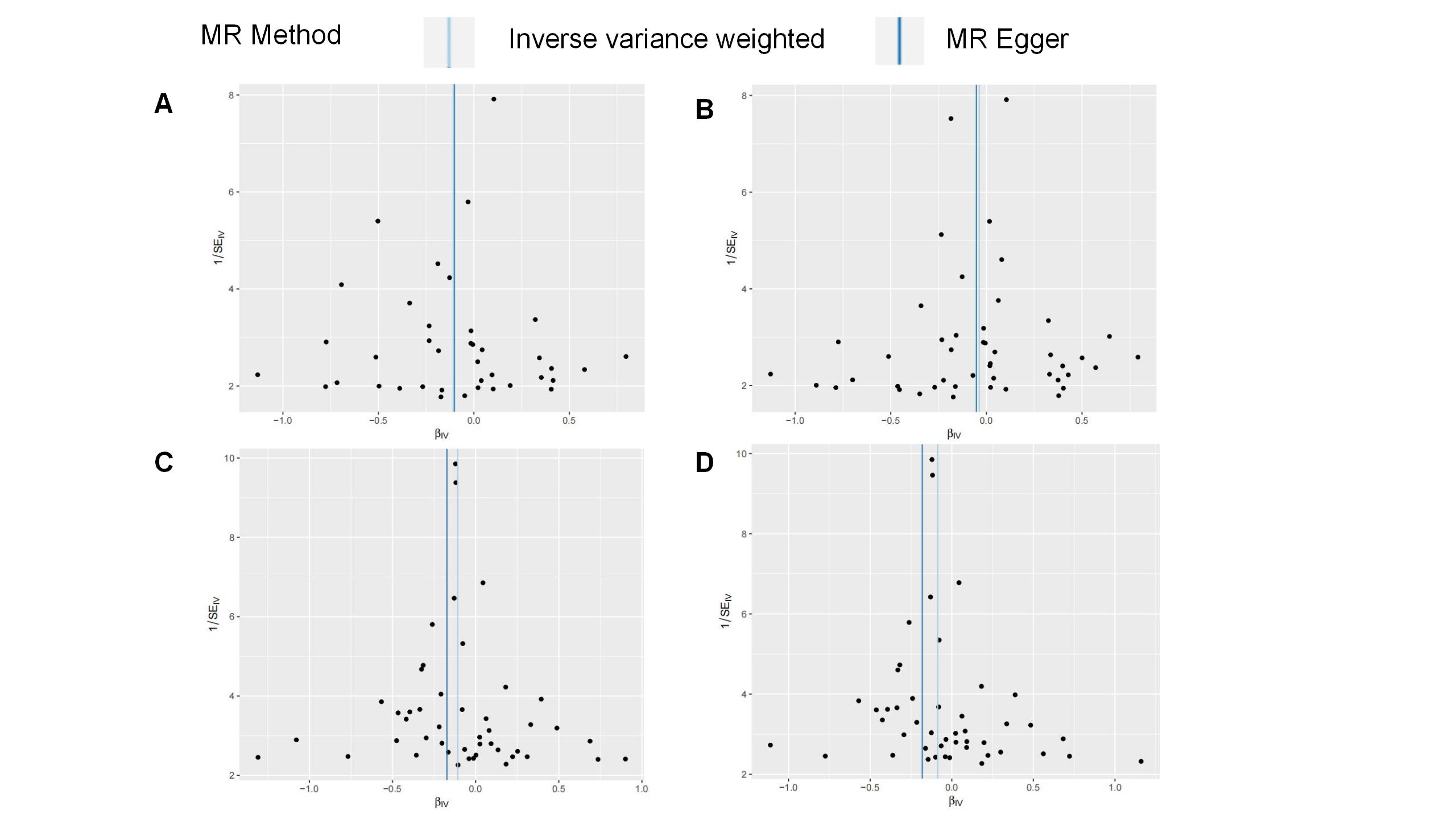


**Supplementary FIGURE 2.** (A-D) Funnel plots for MR analysis between Omega-6s on OA. The x-coordinate is the effect size, and the y-coordinate is the standard error. (A) funnel plot for Omega-6s discovery and HOA; (B) funnel plot for Omega-6s validation and HOA; (C) funnel plot for Omega-6s discovery and KOA; (D) funnel plot for Omega-6s validation and KOA.


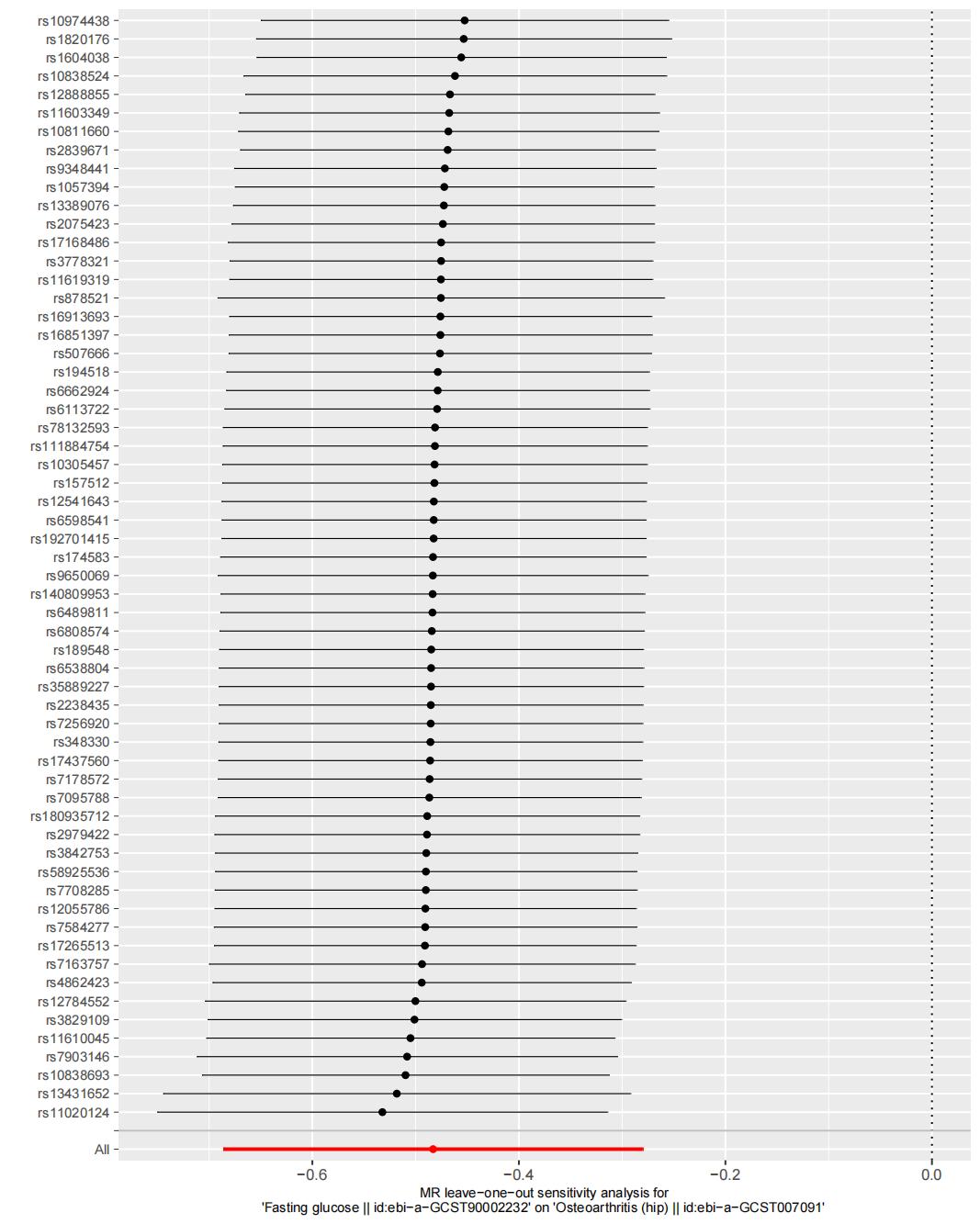


**Supplementary Figure 3A**. Leave-one-out-analysis plot for FG discovery and HOA. The x-coordinate is the combined effect of the remaining SNPs after removing the SNP, and the y-coordinate is the SNP locus.


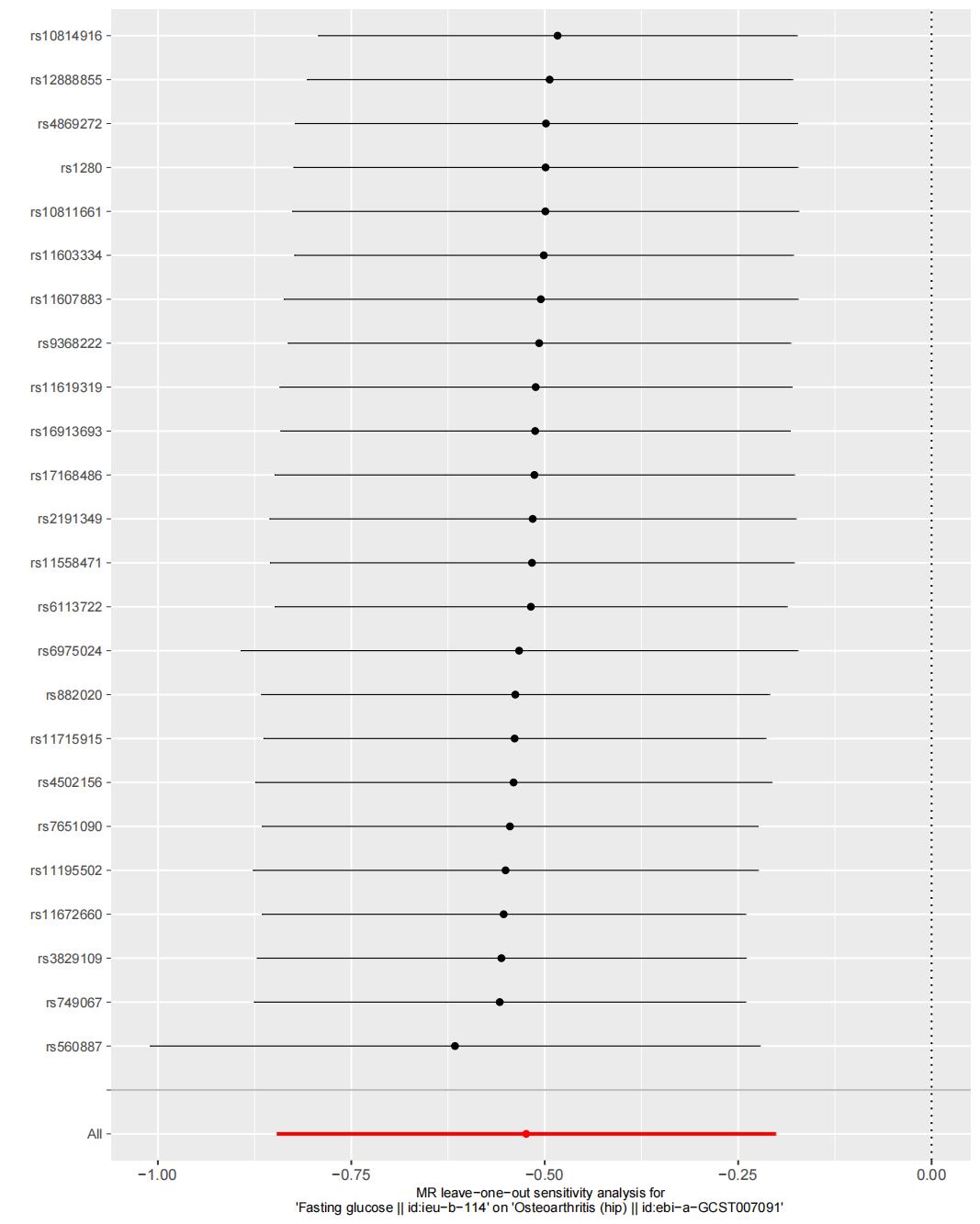


**Supplementary Figure 3B.** Leave-one-out-analysis plot for FG validation and HOA. The x-coordinate is the combined effect of the remaining SNPs after removing the SNP, and the y-coordinate is the SNP locus.


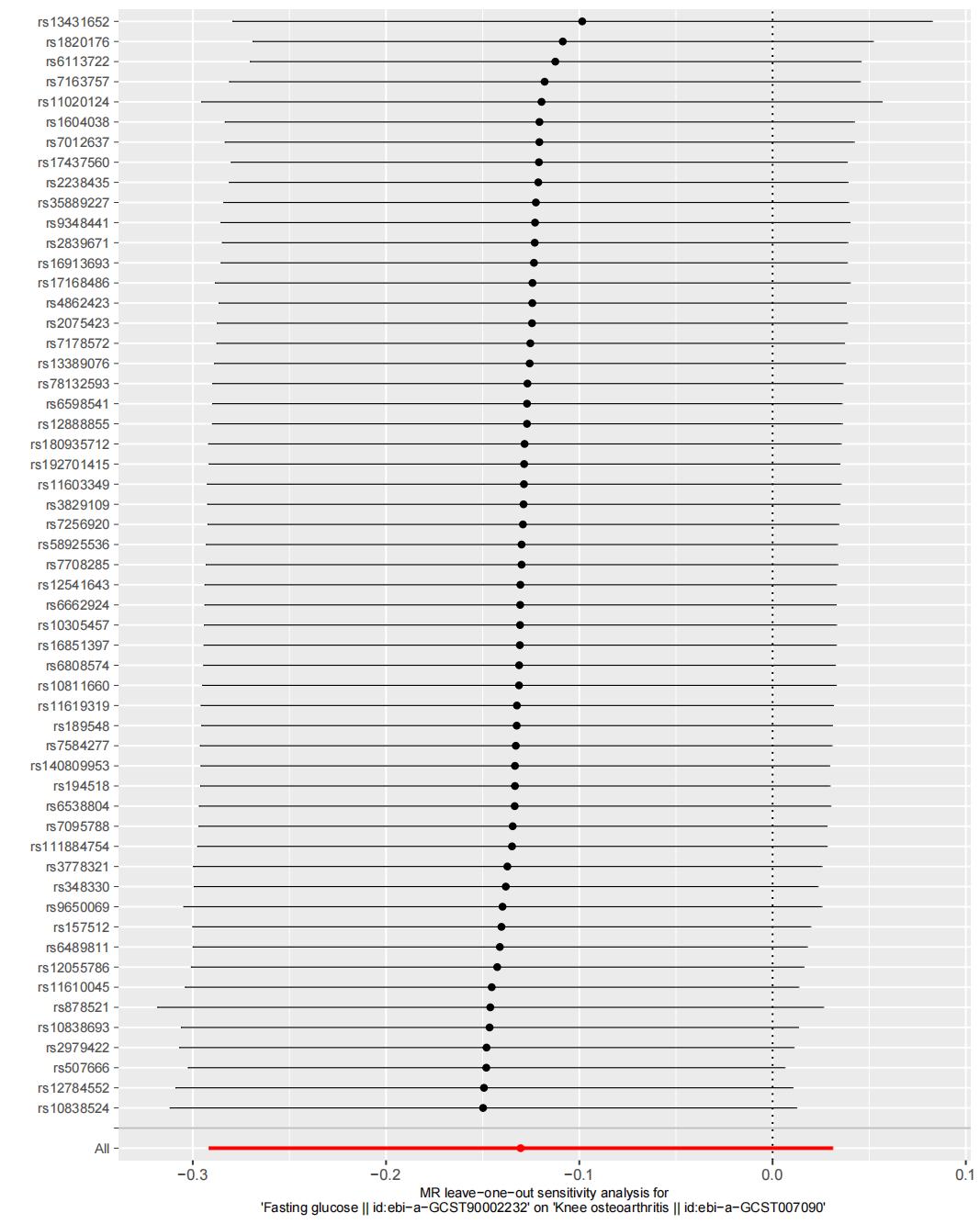


**Supplementary Figure 3C.** Leave-one-out-analysis plot for FG discovery and KOA. The x-coordinate is the combined effect of the remaining SNPs after removing the SNP, and the y-coordinate is the SNP locus.


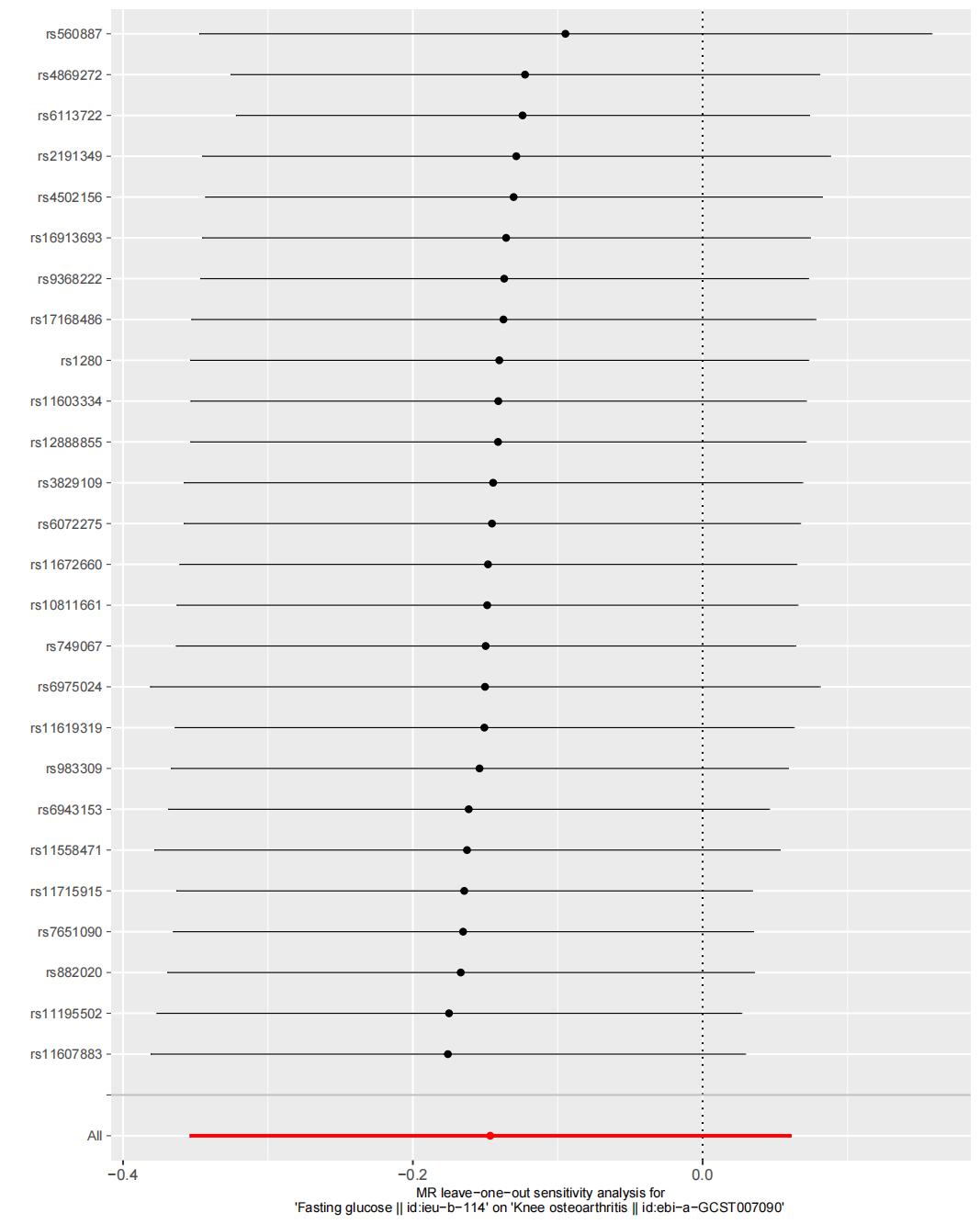


**Supplementary Figure 3D.** Leave-one-out-analysis plot for FG validation and KOA. The x-coordinate is the combined effect of the remaining SNPs after removing the SNP, and the y-coordinate is the SNP locus.


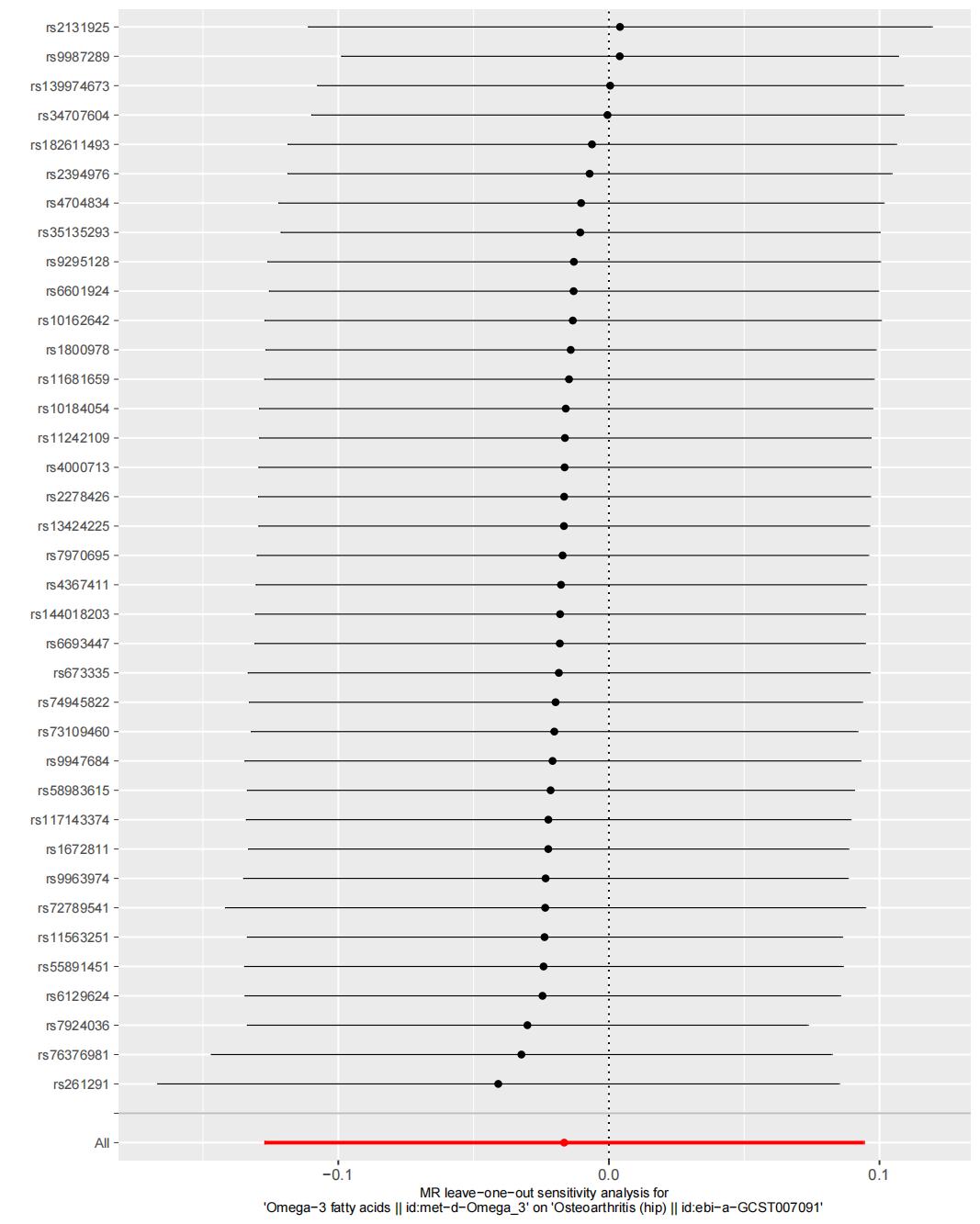


**Supplementary Figure 4A.** Leave-one-out-analysis plot for Omega-3s discovery and HOA. The x-coordinate is the combined effect of the remaining SNPs after removing the SNP, and the y-coordinate is the SNP locus.


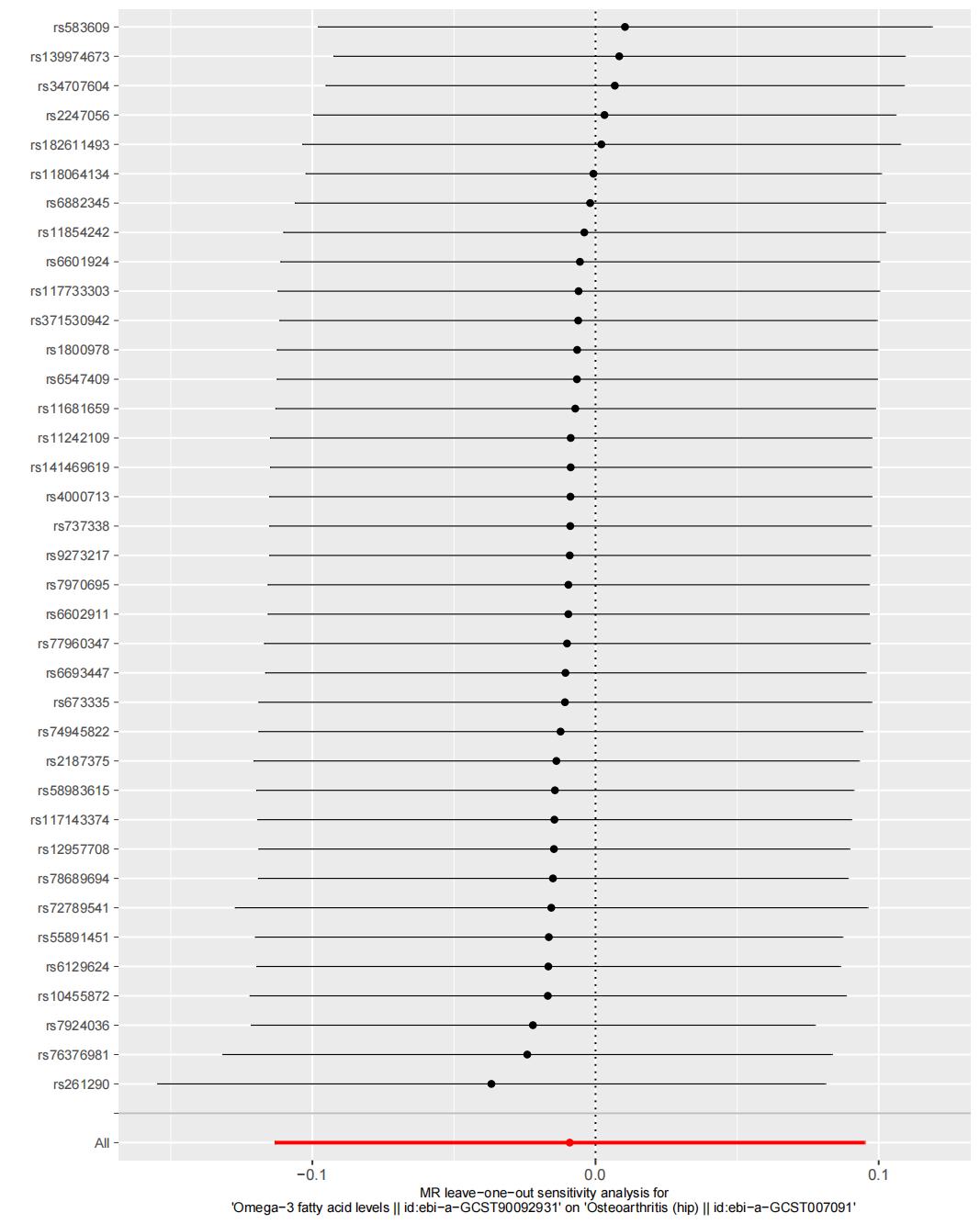


**Supplementary Figure 4B.** Leave-one-out-analysis plot for Omega-3s validation and HOA. The x-coordinate is the combined effect of the remaining SNPs after removing the SNP, and the y-coordinate is the SNP locus.


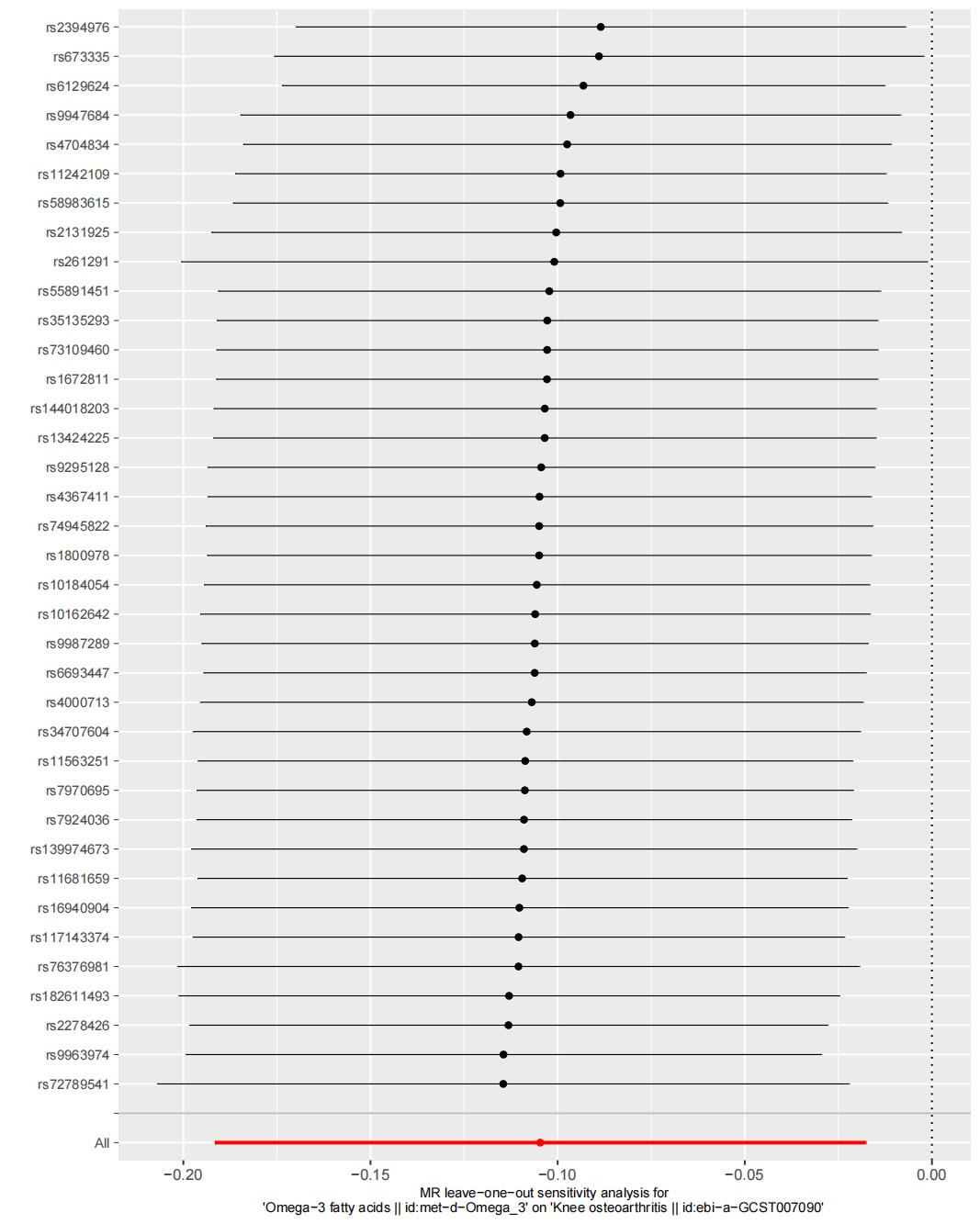


**Supplementary Figure 4C.** Leave-one-out-analysis plot for Omega-3s discovery and KOA. The x-coordinate is the combined effect of the remaining SNPs after removing the SNP, and the y-coordinate is the SNP locus.


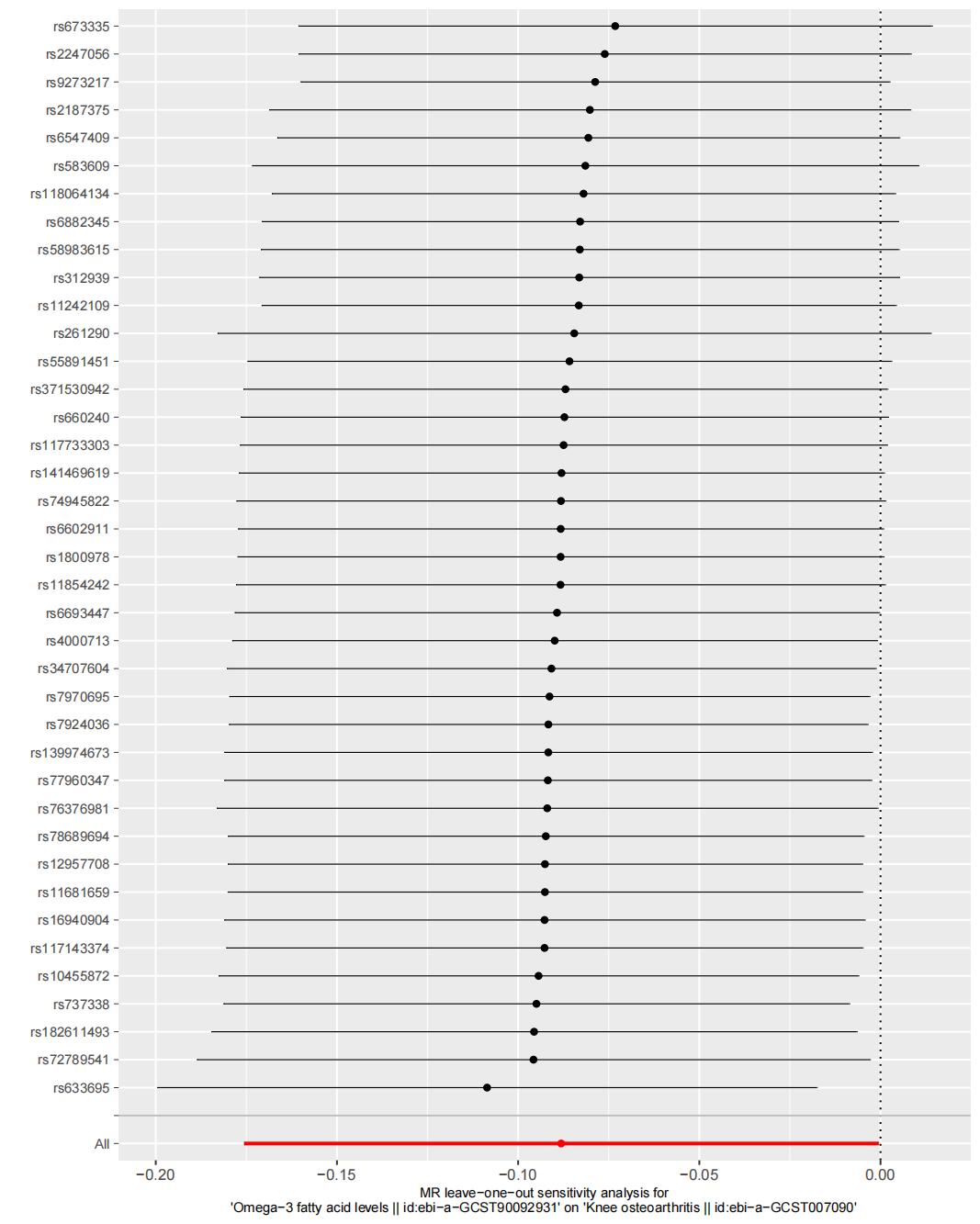


**Supplementary Figure 4D.** Leave-one-out-analysis plot for Omega-3s validation and KOA. The x-coordinate is the combined effect of the remaining SNPs after removing the SNP, and the y-coordinate is the SNP locus.


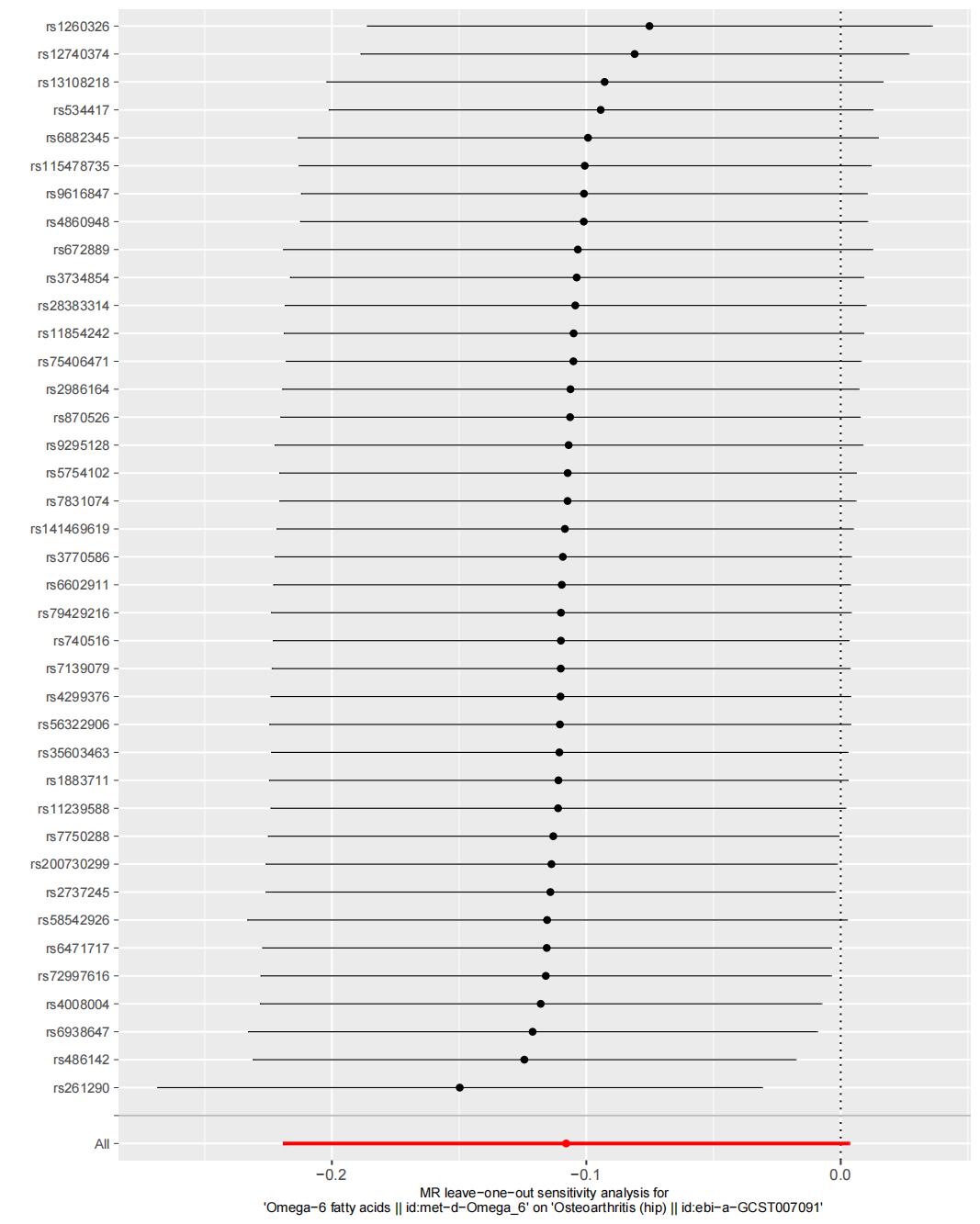


**Supplementary Figure 5A.** Leave-one-out-analysis plot for Omega-6s discovery and HOA. The x-coordinate is the combined effect of the remaining SNPs after removing the SNP, and the y-coordinate is the SNP locus.


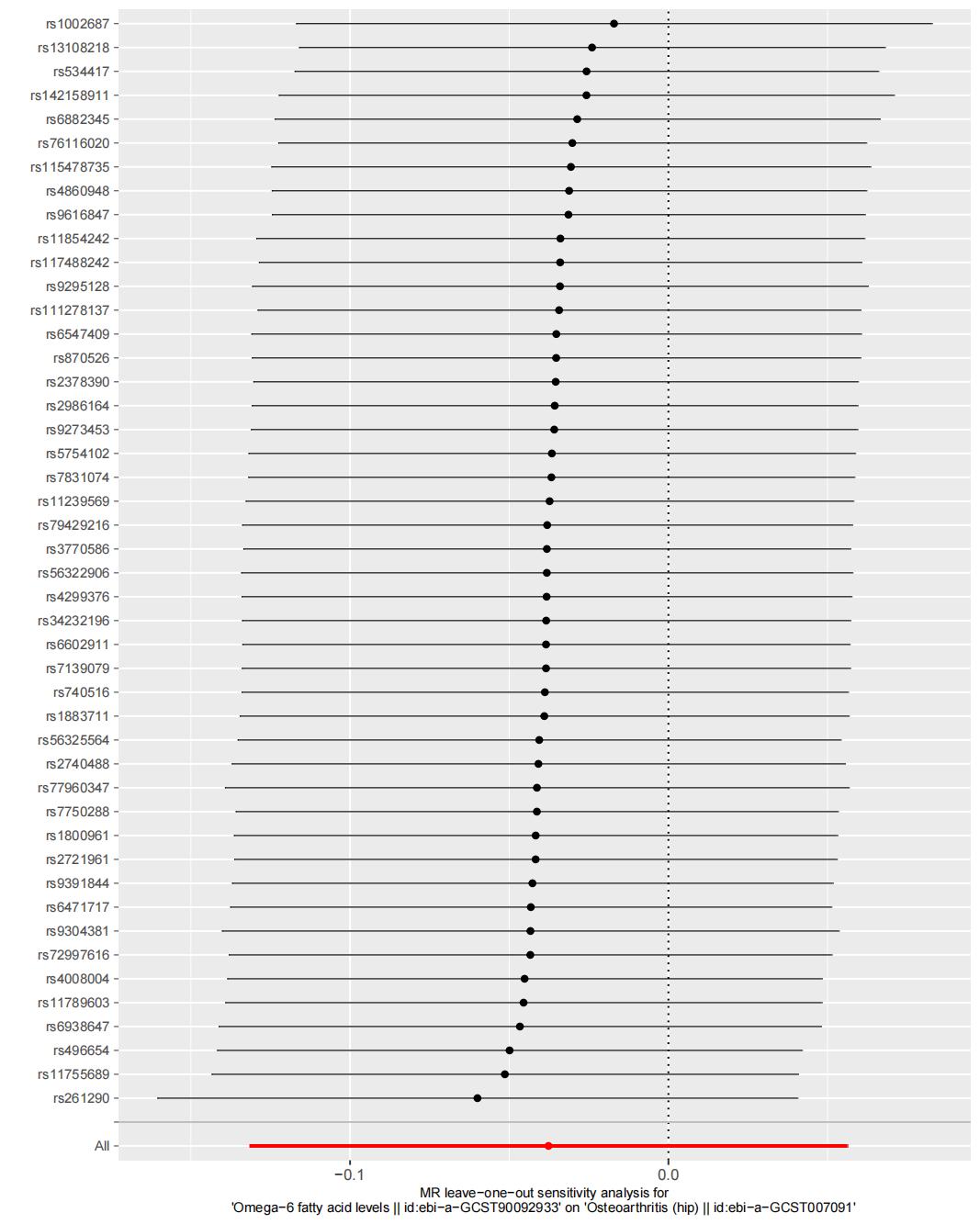


**Supplementary Figure 5B.** Leave-one-out-analysis plot for Omega-6s validation and HOA. The x-coordinate is the combined effect of the remaining SNPs after removing the SNP, and the y-coordinate is the SNP locus.


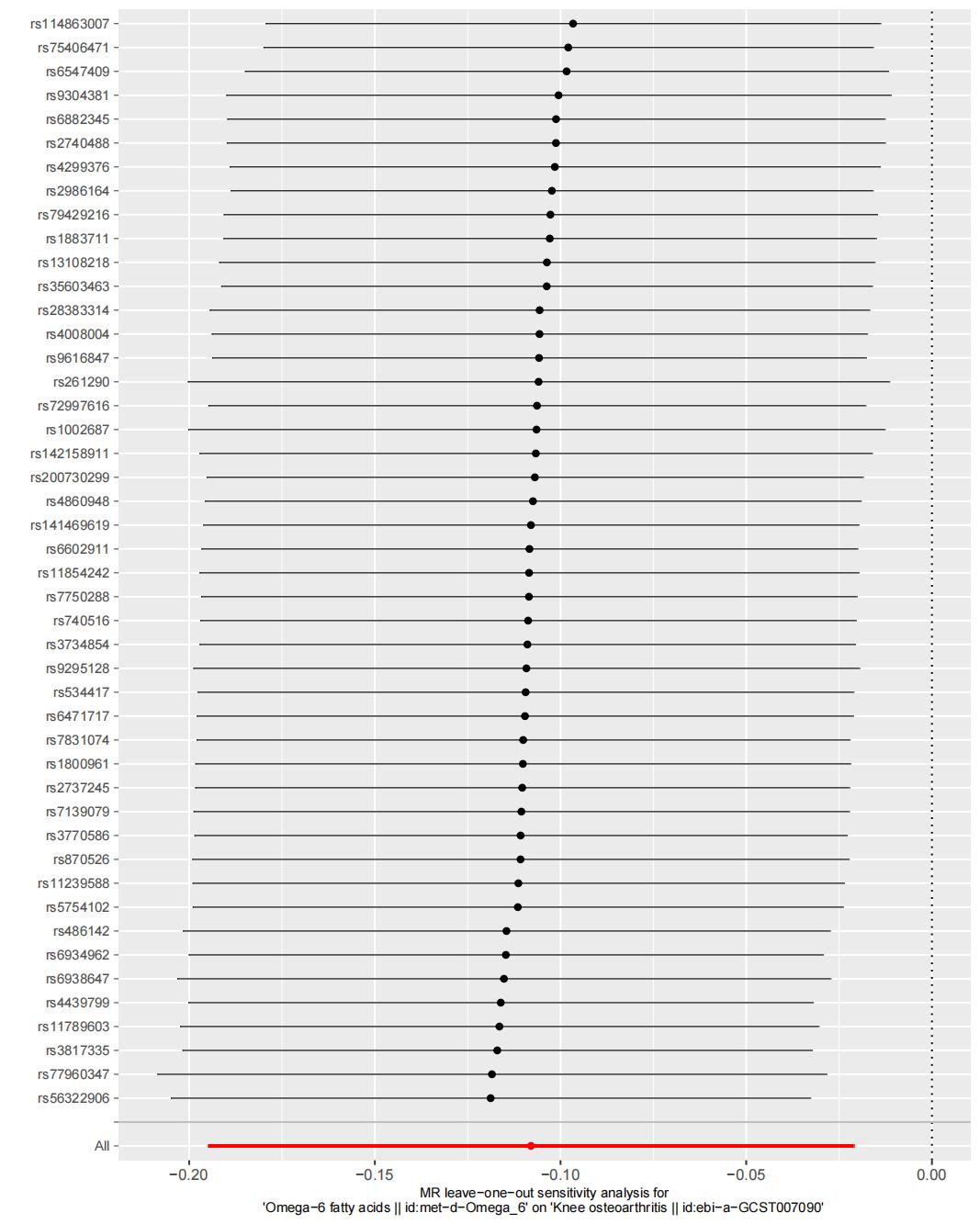


**Supplementary Figure 5C.** Leave-one-out-analysis plot for Omega-6s discovery and KOA. The x-coordinate is the combined effect of the remaining SNPs after removing the SNP, and the y-coordinate is the SNP locus.


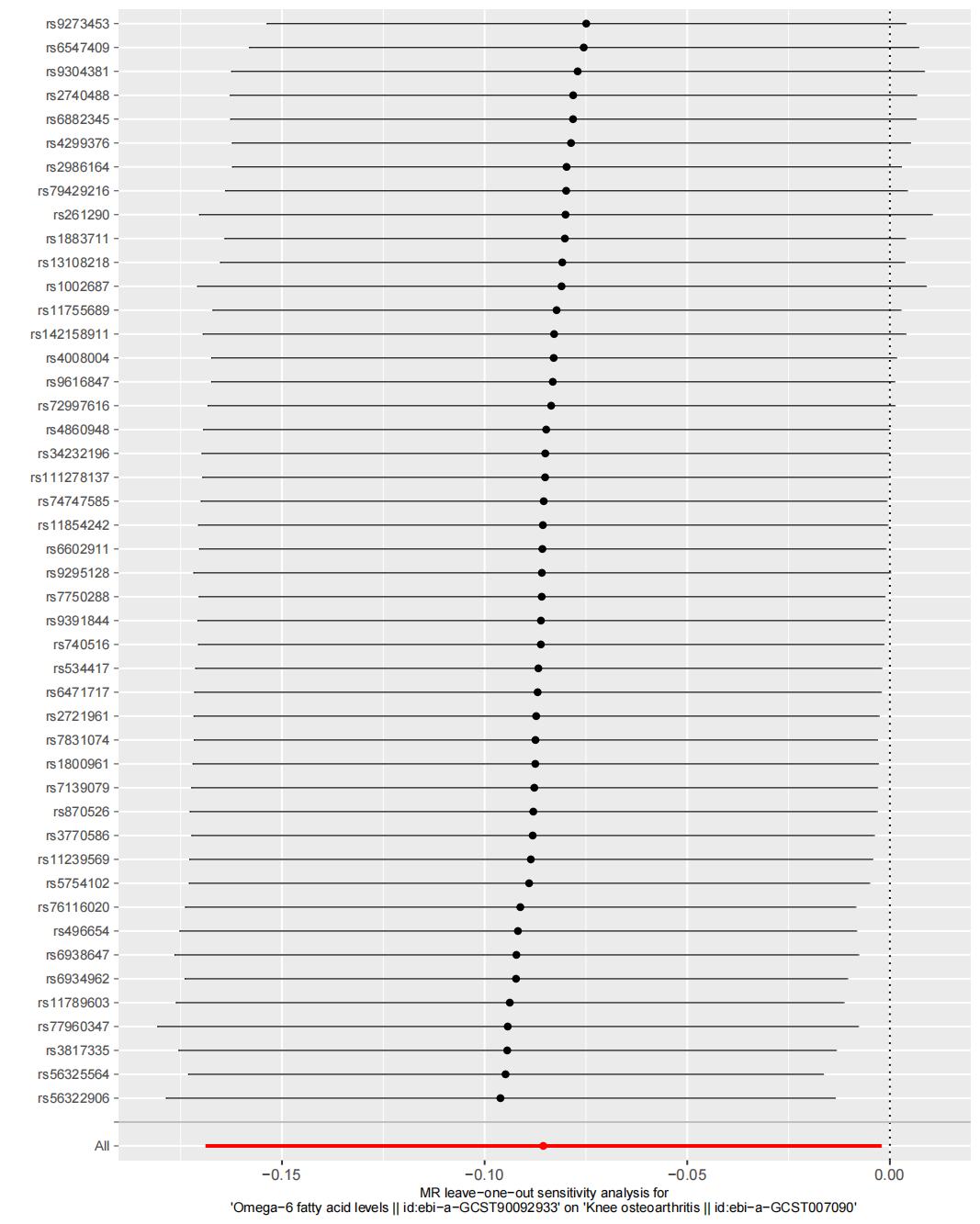


**Supplementary Figure 5D.** Leave-one-out-analysis plot for Omega-6s validation and KOA. The x-coordinate is the combined effect of the remaining SNPs after removing the SNP, and the y-coordinate is the SNP locus.


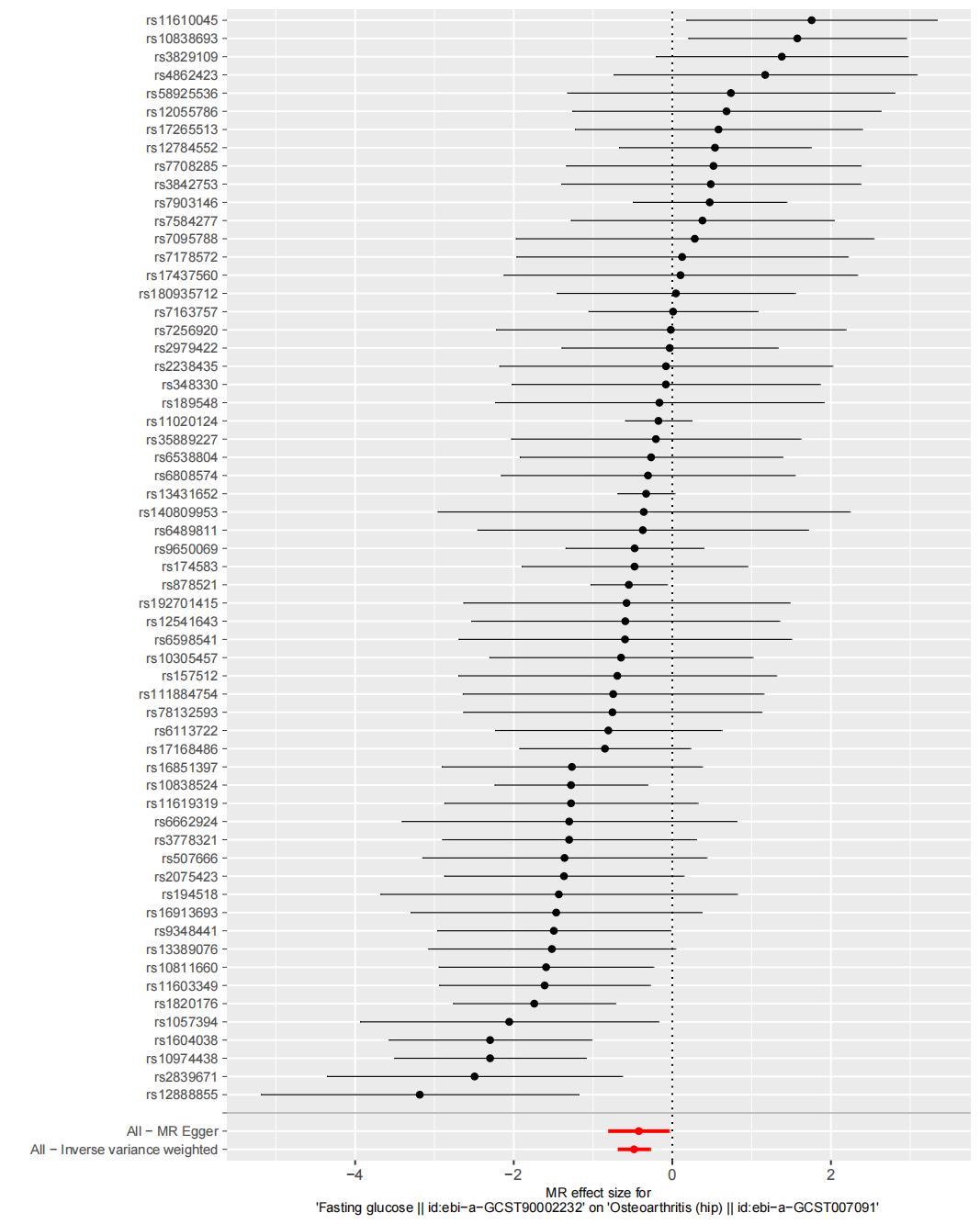


**Supplementary Figure 6A.** Forest plot for FG discovery and HOA. The x-coordinate is the effect size, and the y-coordinate is the SNP locus.


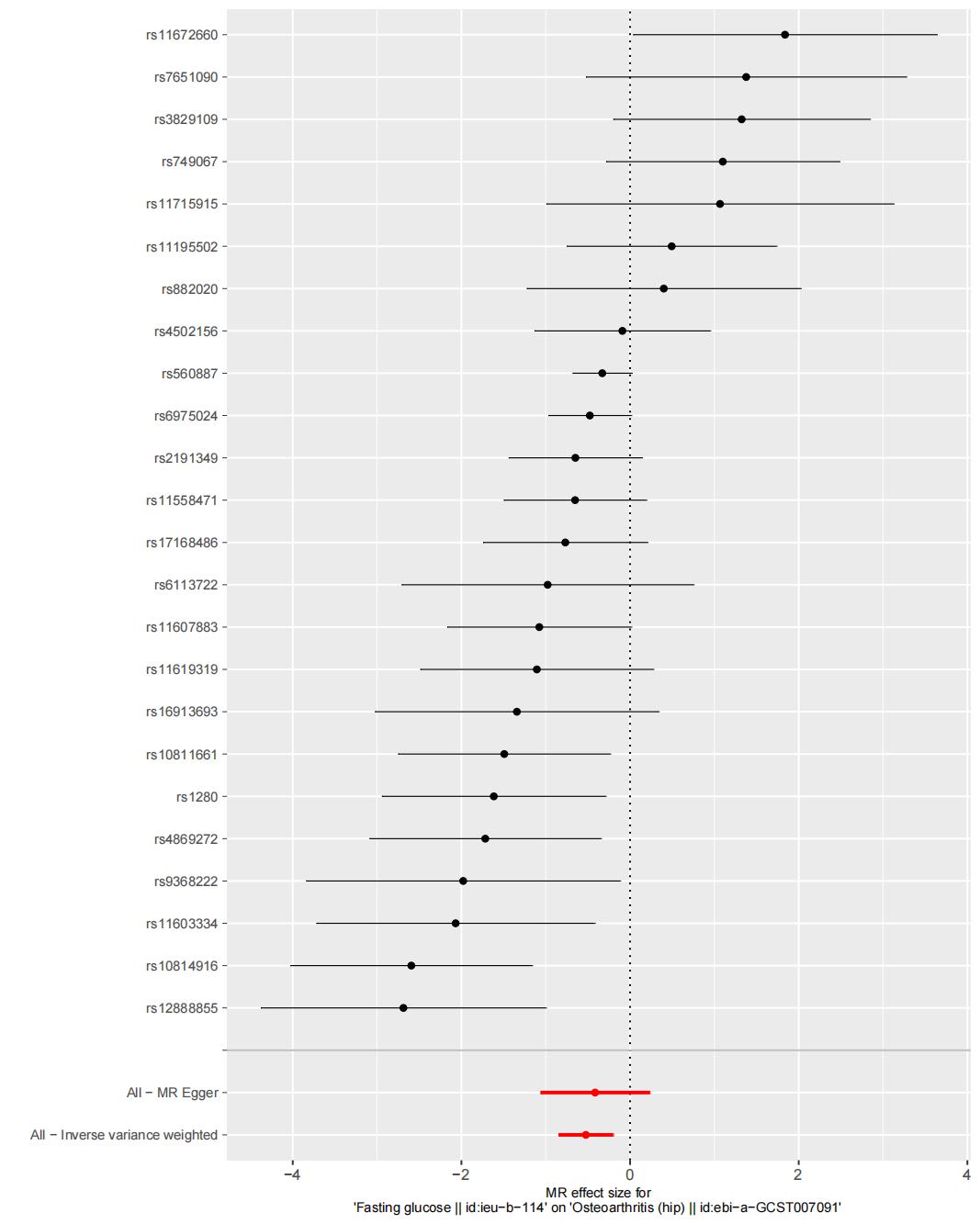


**Supplementary Figure 6B.** Forest plot for FG validation and HOA. The x-coordinate is the effect size, and the y-coordinate is the SNP locus.


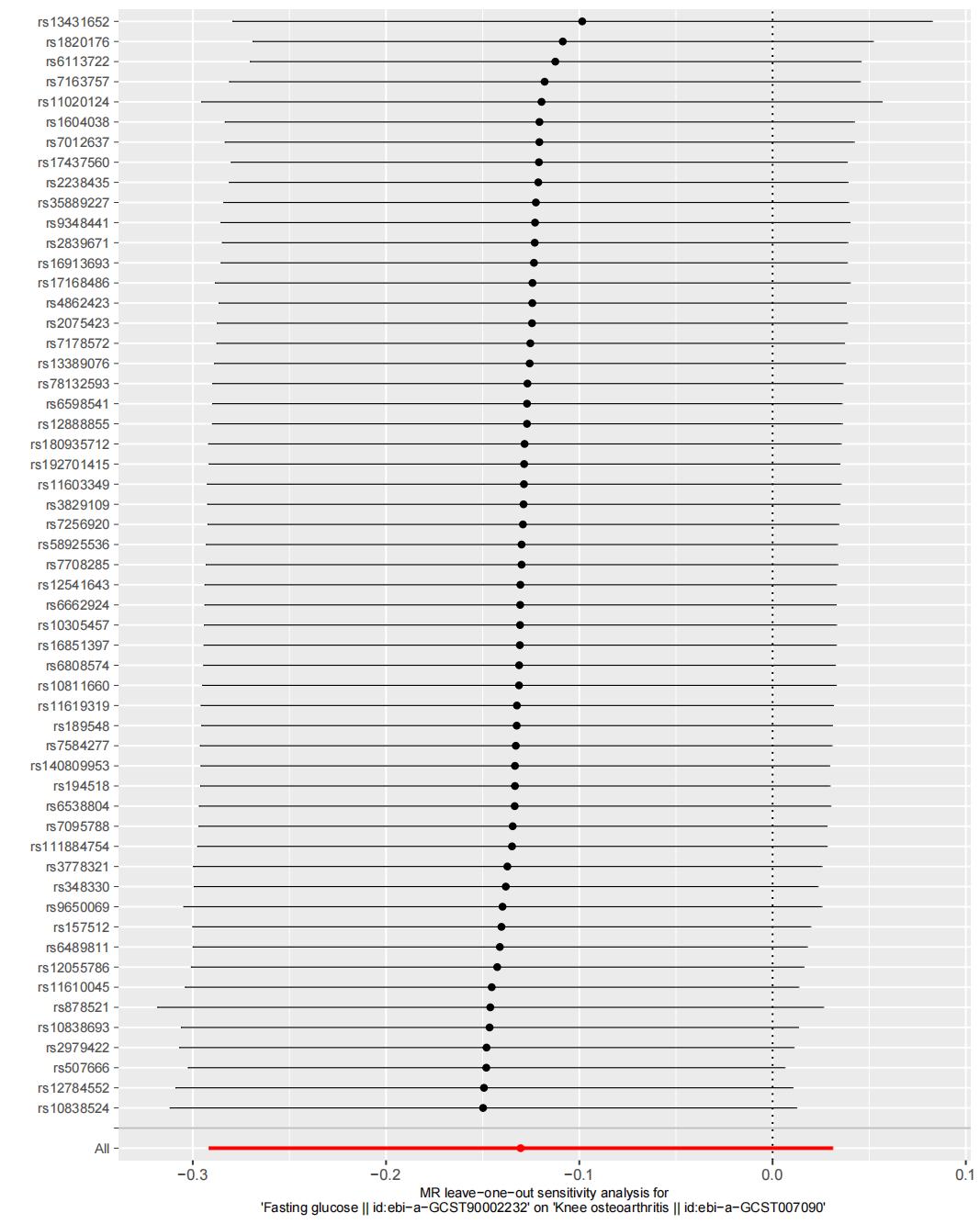


**Supplementary Figure 3C.** Forest plot for FG discovery and KOA. The x-coordinate is the effect size, and the y-coordinate is the SNP locus.


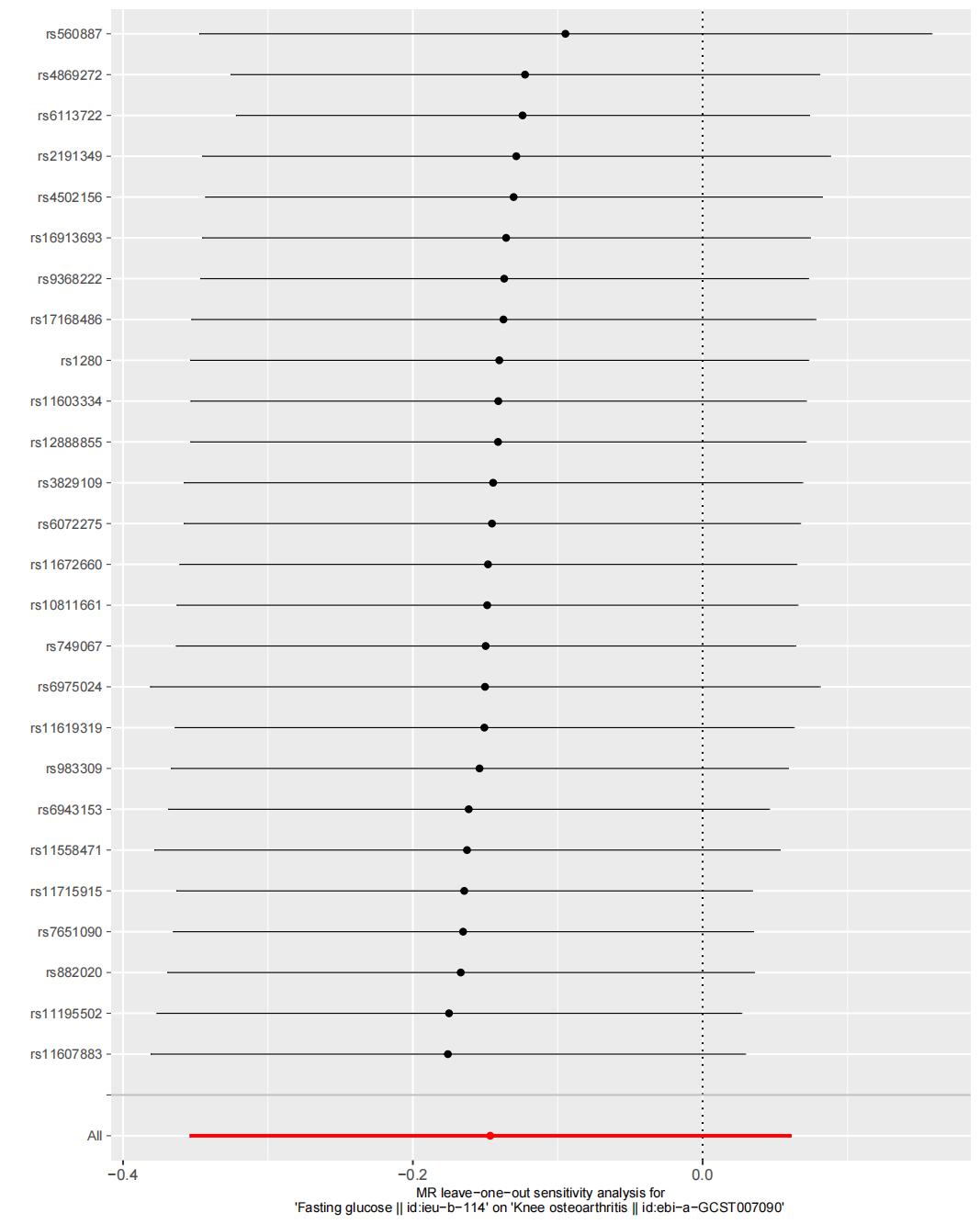


**Supplementary Figure 6D.** Forest plot for FG validation and KOA. The x-coordinate is the effect size, and the y-coordinate is the SNP locus.


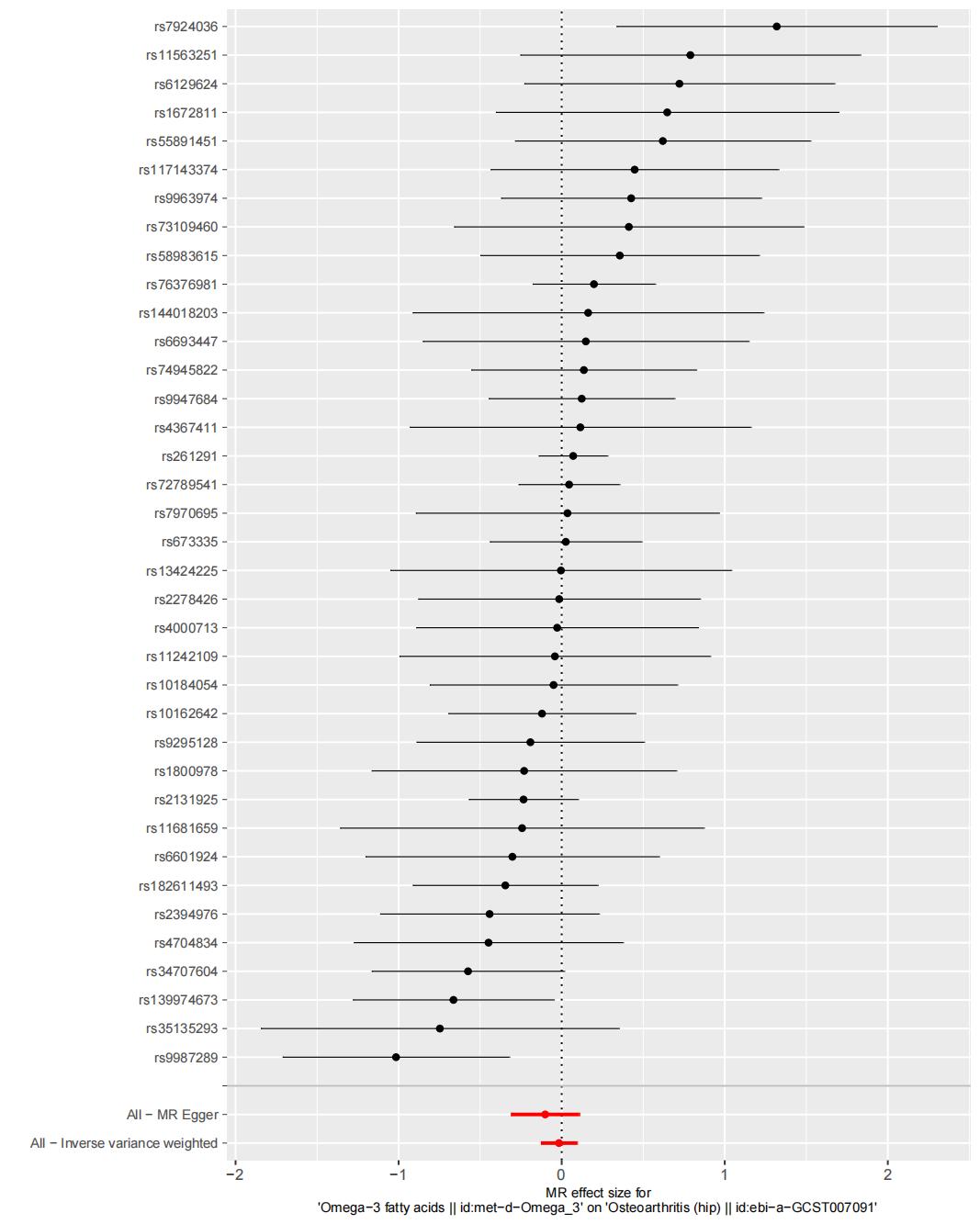


**Supplementary Figure 7A.** Forest plot for Omega-3s discovery and HOA. The x-coordinate is the effect size, and the y-coordinate is the SNP locus.


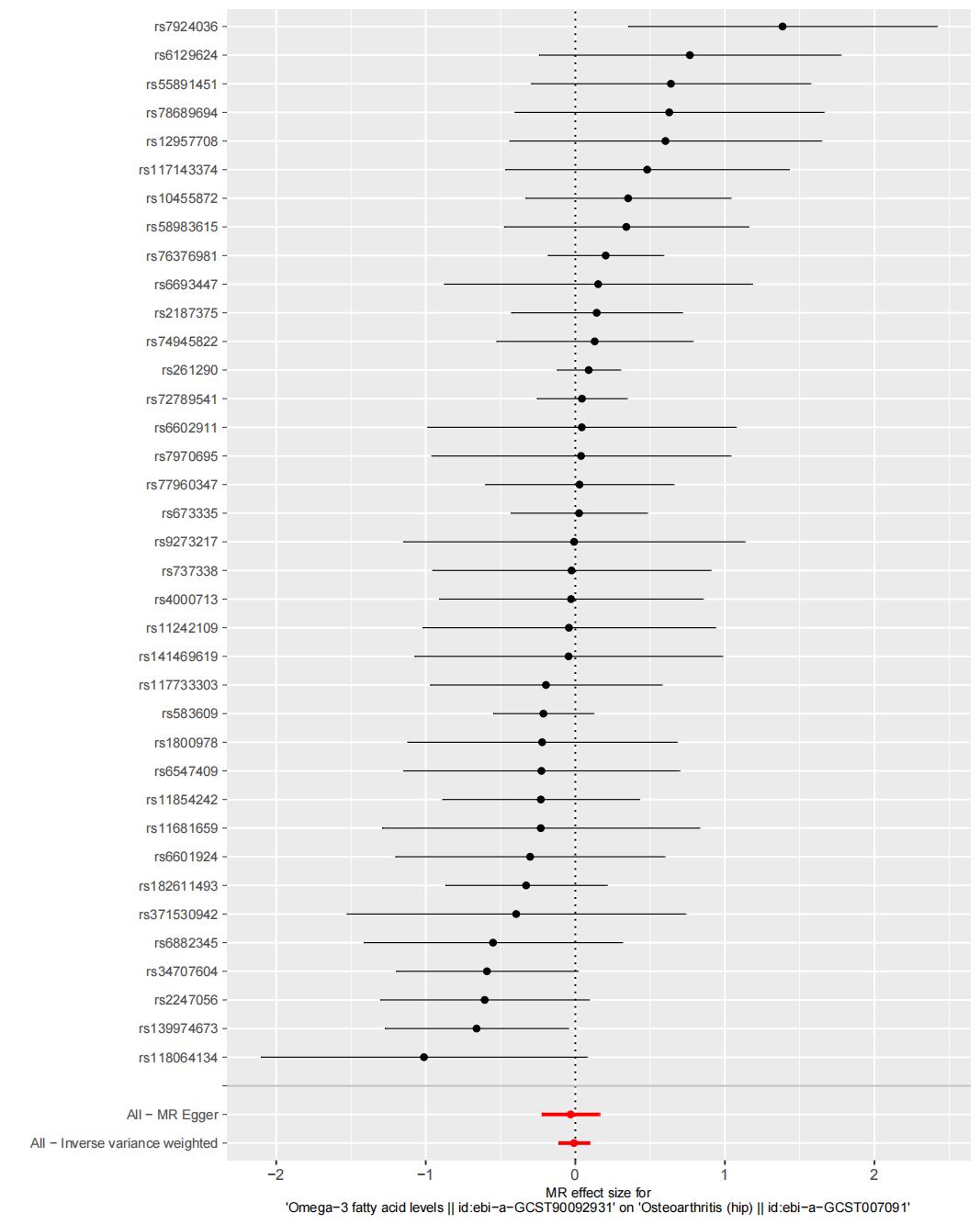


**Supplementary Figure 7B.** Forest plot for Omega-3s validation and HOA. The x-coordinate is the effect size, and the y-coordinate is the SNP locus.


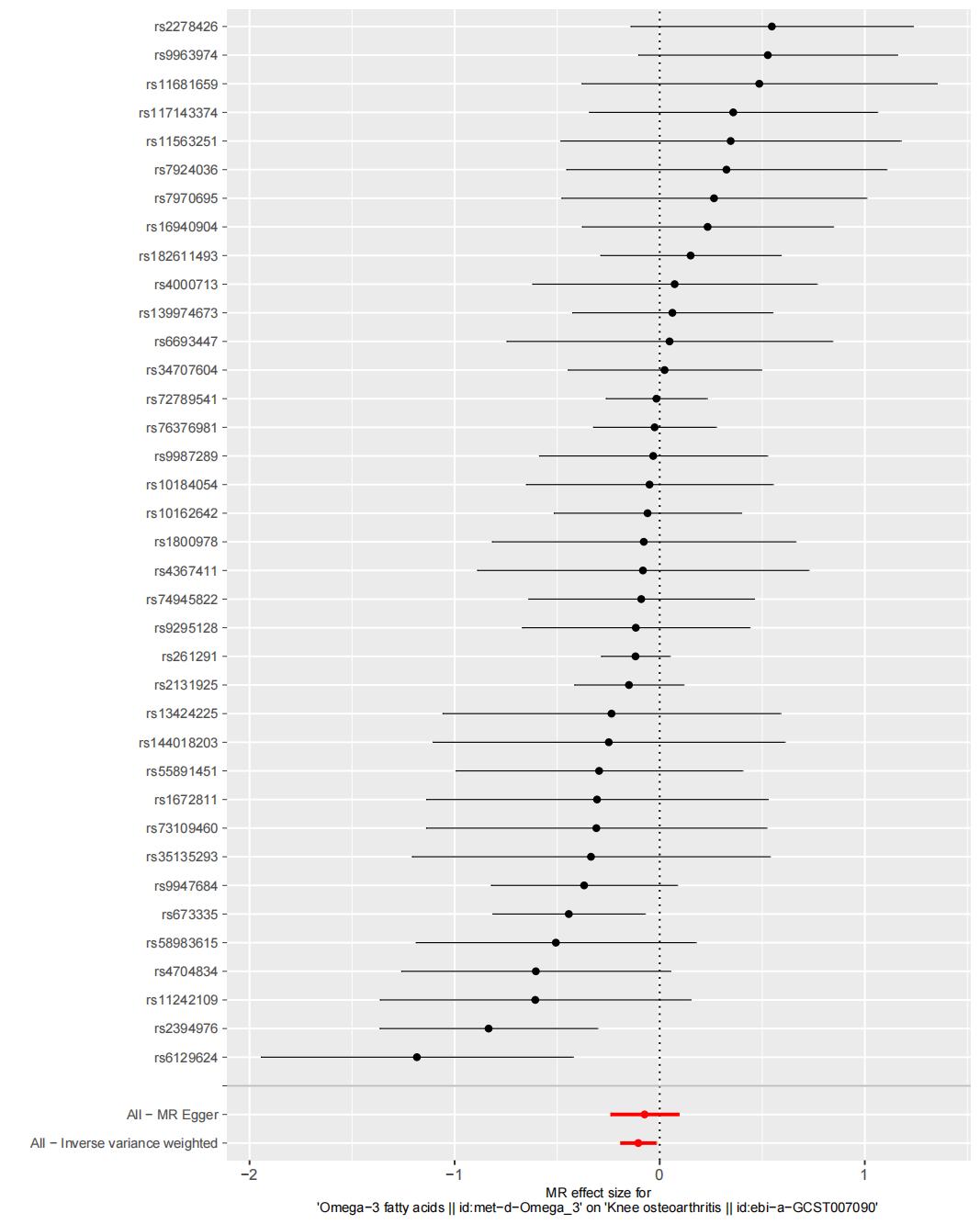


**Supplementary Figure 7C.** Forest plot for Omega-3s discovery and KOA. The x-coordinate is the effect size, and the y-coordinate is the SNP locus.


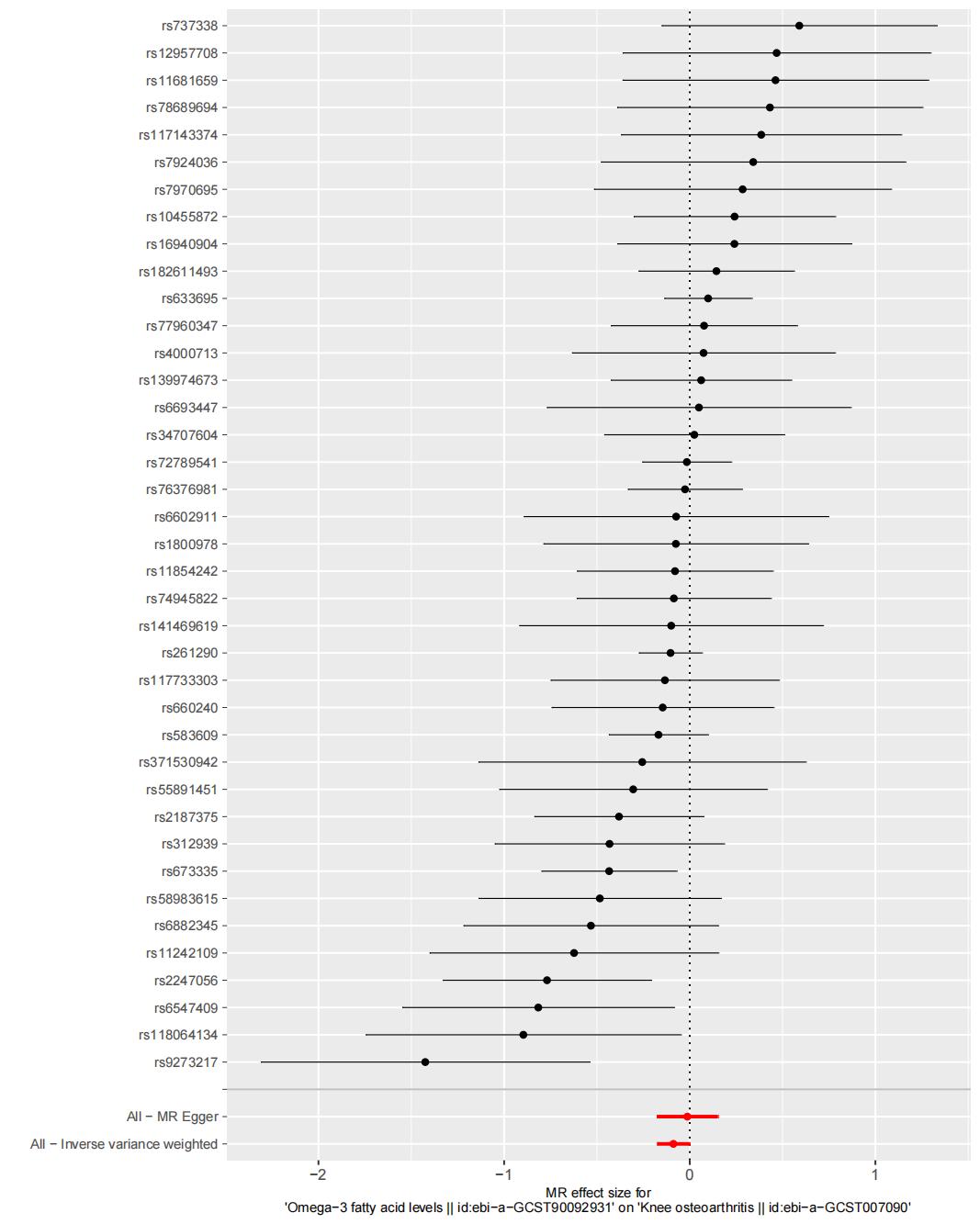


**Supplementary Figure 7D.** Forest plot for Omega-3s validation and KOA. The x-coordinate is the effect size, and the y-coordinate is the SNP locus.


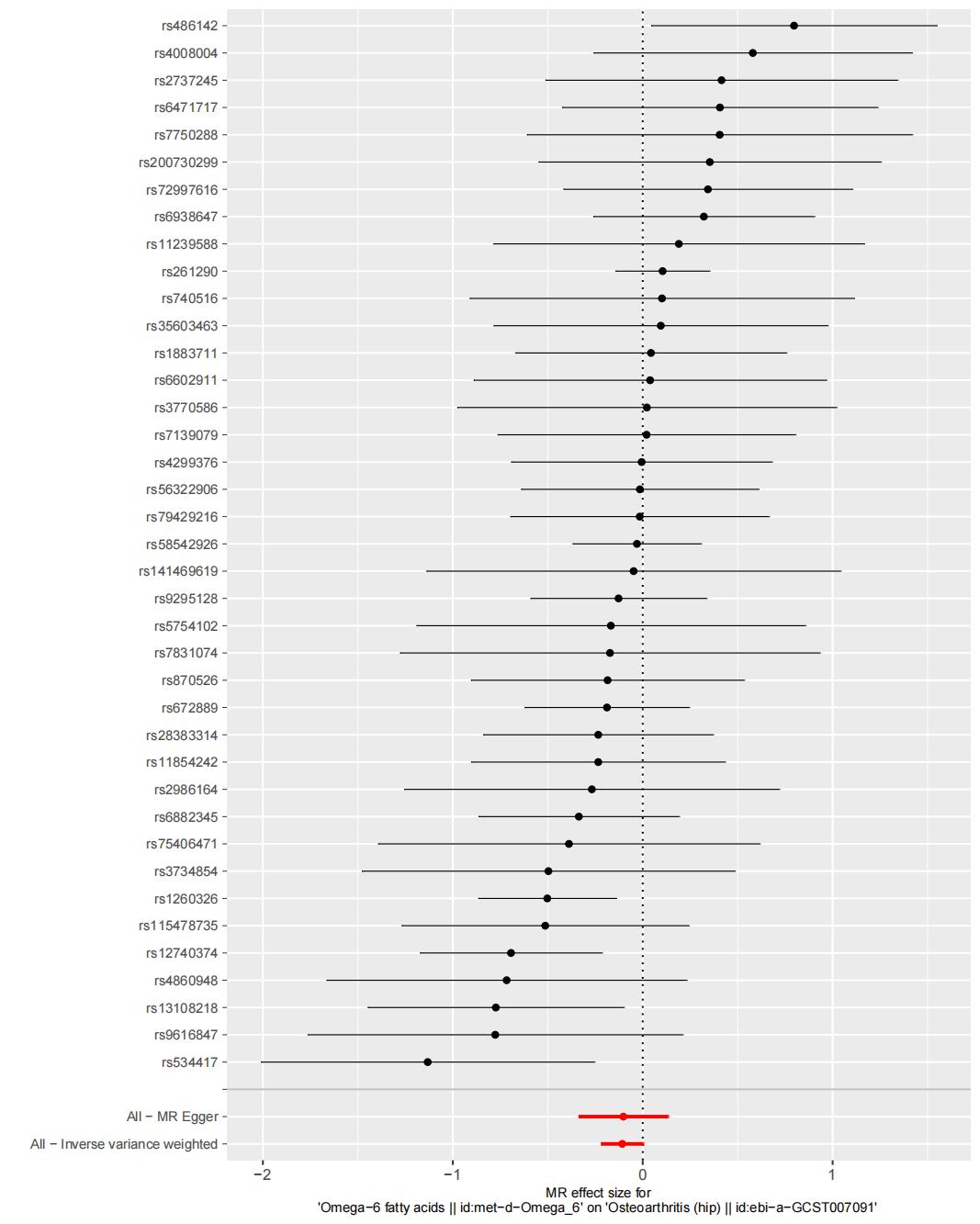


**Supplementary Figure 8A.** Forest plot for Omega-6s discovery and HOA. The x-coordinate is the effect size, and the y-coordinate is the SNP locus.


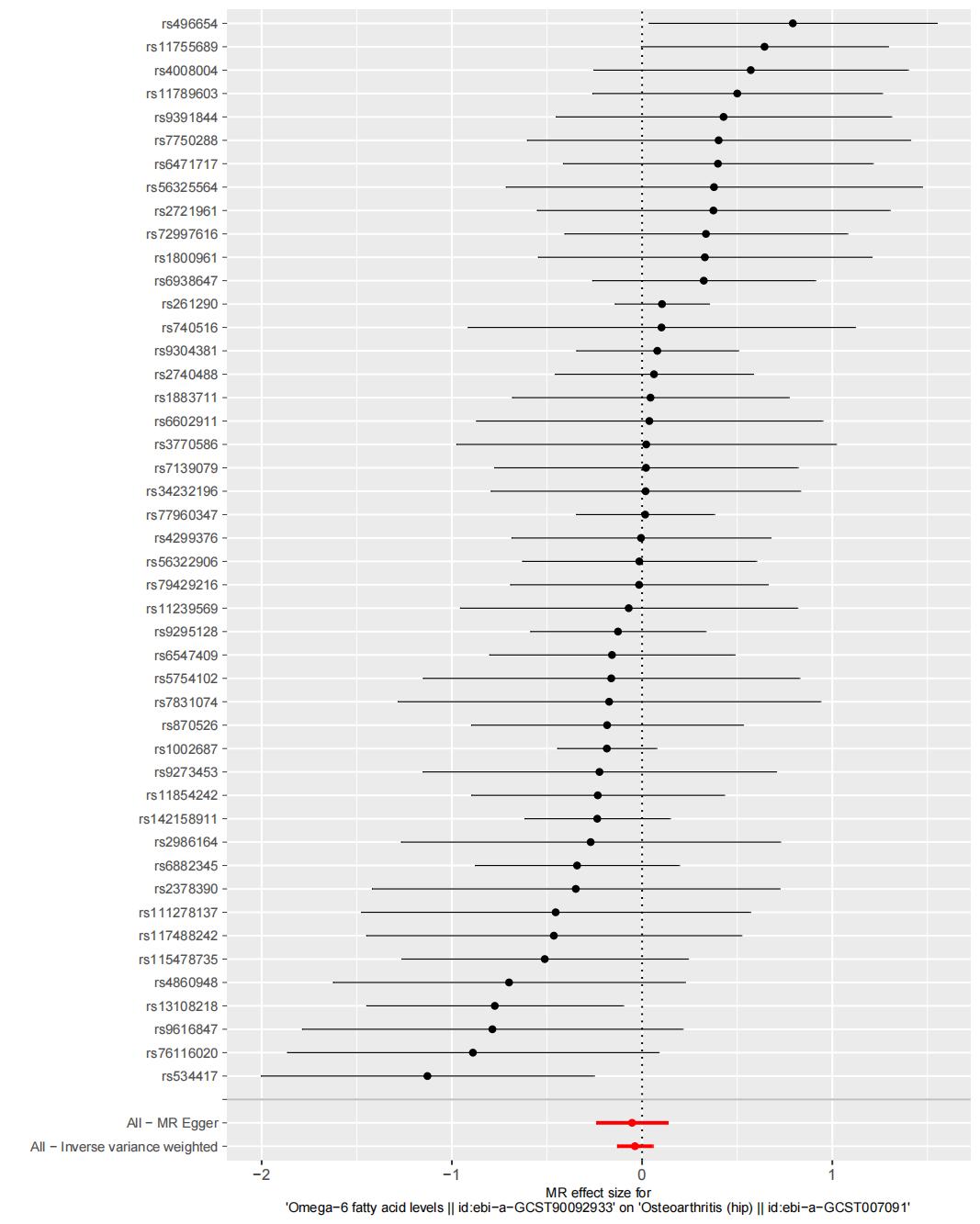


**Supplementary Figure 8B.** Forest plot for Omega-6s validation and HOA. The x-coordinate is the effect size, and the y-coordinate is the SNP locus.


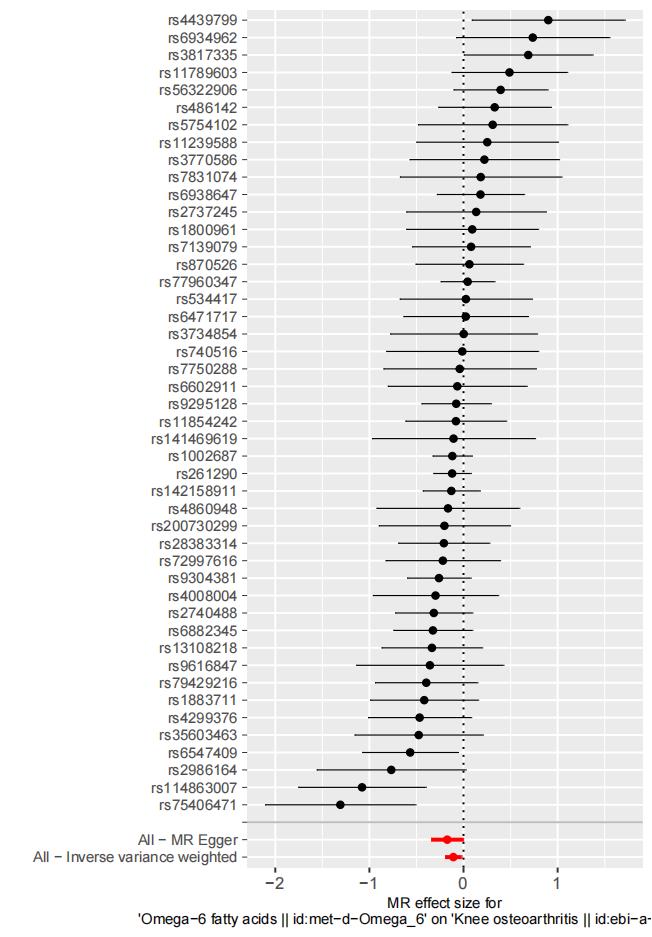


**Supplementary Figure 8C.** Forest plot for Omega-6s discovery and KOA. The x-coordinate is the effect size, and the y-coordinate is the SNP locus.


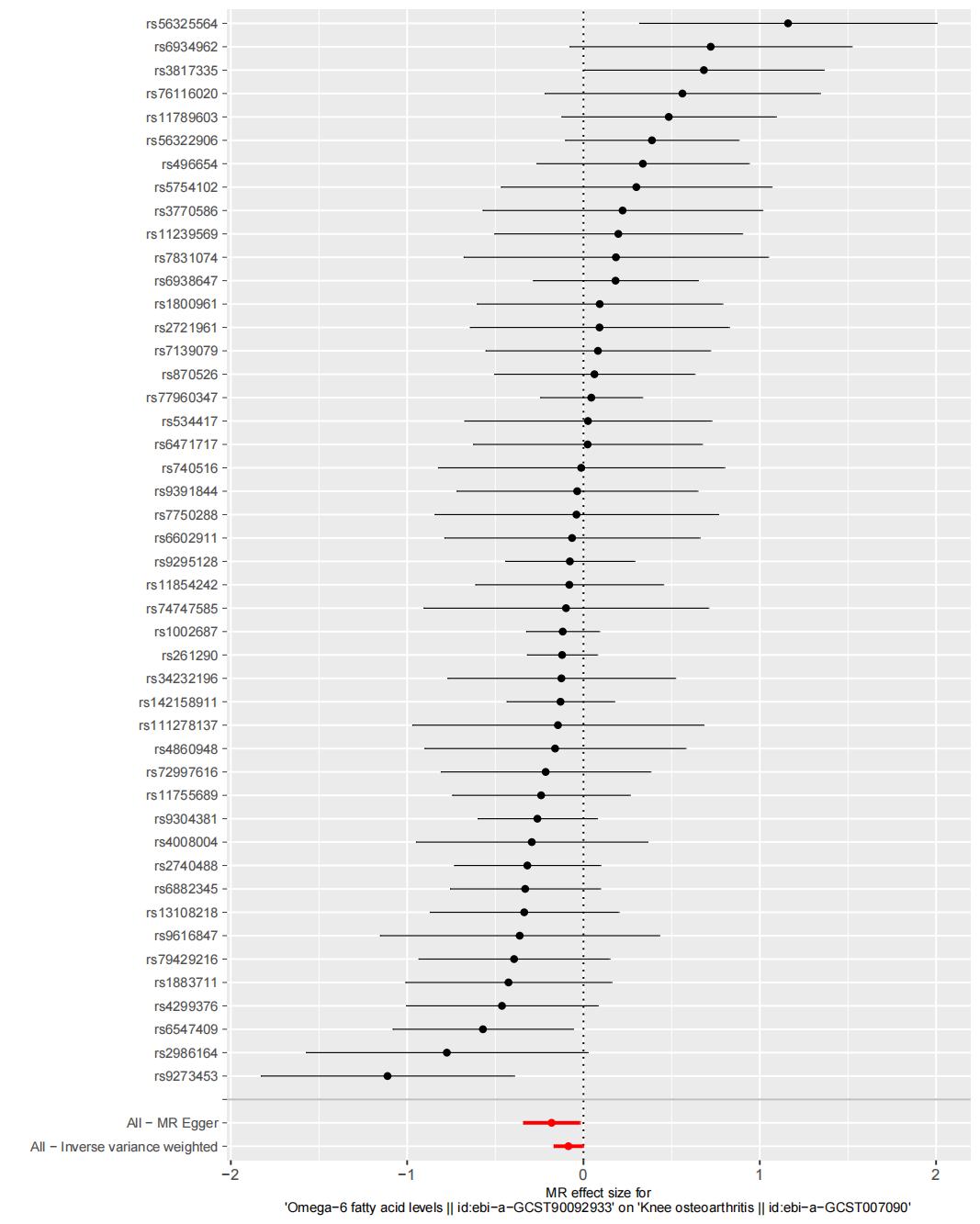


**Supplementary Figure 8D.** Forest plot for Omega-6s validation and KOA. The x-coordinate is the effect size, and the y-coordinate is the SNP locus.

1.2 Supplementary Tables.

Table 1. MVMR result between OA and metabolic related factors.

| Exposure | Outcome | SNPs number | IVW method | |
| --- | --- | --- | --- | --- |
|  |  |  | OR (95%CI) | P value |
| FG discovery | HOA | 18 | 0.79(0.61~1.02) | 0.068  0.79(0.61~1.02)  23 |
| Omega-3s discovery | HOA | 9 | 1.01(0.92~1.12) | 0.774 |
| Omega-6s discovery | HOA | 11 | 0.92(0.74~1.16) | 0.494 |
| BMI | HOA | 135 | 1.56(1.33~1.82) | < 0.001 |
| SHBG | HOA | 177 | 1.02(0.94~1.10) | 0.675 |
| FG discovery | KOA | 17 | 0.79(0.64~0.99) | 0.036 |
| Omega-3s discovery | KOA | 11 | 1.03(0.95~1.12) | 0.473 |
| Omega-6s discovery | KOA | 13 | 0.89(0.76~1.04) | 0.143 |
| BMI | KOA | 134 | 1.86(1.63~2.11) | < 0.001 |
| SHBG | KOA | 176 | 0.96(0.90~1.02) | 0.235 |

Note: FG, fasting glucose; Omega-3s, omega-3 fatty acids; Omega-6s, omega-6 fatty acids; HOA, hip osteoarthritis; KOA, knee osteoarthritis; BMI, body mass index; SHBG, Sex hormone-binding globulin levels; OR, odds ratio.
